# The m6A reader IGF2BP3 preserves NOTCH3 mRNA stability to sustain Notch3 signaling and promote tumor metastasis in nasopharyngeal carcinoma

**DOI:** 10.1038/s41388-023-02865-6

**Published:** 2023-10-18

**Authors:** Boyu Chen, Runda Huang, Tianliang Xia, Chunyang Wang, Xiao Xiao, Shunzhen Lu, Xiangfu Chen, Ying Ouyang, Xiaowu Deng, Jingjing Miao, Chong Zhao, Lin Wang

**Affiliations:** 1 State Key Laboratory of Oncology in South China, Guangdong Key Laboratory of Nasopharyngeal Carcinoma Diagnosis and Therapy, Guangdong Provincial Clinical Research Center for Cancer, Sun Yat-sen University Cancer Center, Guangzhou, 510060 P. R. China; 2 Department of Experimental Research, Sun Yat-sen University Cancer Center, Guangzhou, P. R. China; 3 Department of Radiation Oncology, Sun Yat-sen University Cancer Center, Guangzhou, P. R. China; 4 Guanghua School of Stomatology, Hospital of Stomatology, Guangdong Provincial Key Laboratory of Stomatology, Sun Yat-sen University, Guangzhou, 510060 P. R. China; 5 Department of Nasopharyngeal Carcinoma, Sun Yat-sen University Cancer Center, Guangzhou, P. R. China

**Keywords:** Head and neck cancer, Cell signalling, Metastasis

## Abstract

Metastasis remains the major cause of treatment failure in patients with nasopharyngeal carcinoma (NPC), in which sustained activation of the Notch signaling plays a critical role. N6-Methyladenosine (m6A)-mediated post-transcriptional regulation is involved in fine-tuning the Notch signaling output; however, the post-transcriptional mechanisms underlying NPC metastasis remain poorly understood. In the present study, we report that insulin-like growth factor 2 mRNA-binding proteins 3 (IGF2BP3) serves as a key m6A reader in NPC. IGF2BP3 expression was significantly upregulated in metastatic NPC and correlated with poor prognosis in patients with NPC. IGF2BP3 overexpression promoted, while IGF2BP3 downregulation inhibited tumor metastasis and the stemness phenotype of NPC cells in vitro and in vivo. Mechanistically, IGF2BP3 maintains NOTCH3 mRNA stability via suppression of CCR4-NOT complex-mediated deadenylation in an m6A-dependent manner, which sustains Notch3 signaling activation and increases the transcription of stemness-associated downstream genes, eventually promoting tumor metastasis. Our findings highlight the pro-metastatic function of the IGF2BP3/Notch3 axis and revealed the precise role of IGF2BP3 in post-transcriptional regulation of NOTCH3, suggesting IGF2BP3 as a novel prognostic biomarker and potential therapeutic target in NPC metastasis.

## Introduction

Nasopharyngeal carcinoma (NPC) is a metastasis-prone malignant tumor arising from the nasopharyngeal epithelium, in which approximately 70% of patients present with locoregionally advanced NPC at first diagnosis [1–3]. Despite progress in systemic treatment based on radiotherapy breakthroughs improving treatment outcomes, 20–30% of patients still developed distant metastases, which contribute to the majority deaths of patients with NPC [4, 5]. Hence, there is an urgent need to identify the molecular mechanisms underlying NPC tumor metastasis.

The development of metastasis is a highly inefficient multistage process, in which organ colonization is regarded as the most complicated and rate-limiting step [6]. Disseminated tumor cells (DTCs) possessing stemness traits are a prerequisite to the successful metastatic colony formation and critical for the initial expansion in this early phase of metastatic colonization [7, 8]. The Notch signaling pathway have been demonstrated that are widely overactivated and regulates stemness and metastasis in multiple cancers [9–12]. Multiple mechanisms fine-tune the Notch signaling output, in which post-transcriptional modification might be essential and potentially develop as a novel therapeutic avenue [9, 13–16]. Moreover, it has been demonstrated that the suppression of the Notch signaling pathway reduced the proliferation and enhanced the treatment sensitivity of NPC cells [17–19]. However, how post-transcriptional modification regulates the Notch pathway in NPC are incompletely understood.

N6-Methyladenosine (m6A) is one of the post-transcriptional types of multiple cancers [20]. The global abundance of m6A in cancer cells is critical for cancer initiation, progression, metastasis, relapse and treatment resistance [21], which is regulated by m6A writers, including methyltransferase Like 3 (METTL3), METTL14 and WT1 associated protein (WTAP), and m6A erasers, such as fat mass and obesity associated protein (FTO) and AlkB homolog (ALKBH). Notably, m6A readers mediate effects of m6A modification by associating m6A-modified RNAs with mRNA processing enzymes and eventually control the fate of the modified RNAs [20]. YTH domain family proteins, such as YTHDF1, YTHDF2, and YTHDF3, regulates the stability of m6A-bearing transcripts in the cytoplasm to increase tumor malignancy [22, 23]. Heterogeneous nuclear ribonucleoproteins, including HNRNPA2/B1, HNRNPC, and HNRNPG, regulate alternative splicing or processing of target transcripts in nucleus [24, 25]. Moreover, insulin-like growth factor 2 mRNA-binding proteins 1–3 (IGF2BP1–3), are a newly reported family of m6A readers that prevent m6A-modified mRNAs decay [26, 27]. Aberrant expression of the reader proteins plays an essential role in cancer metastasis [28]. However, the precise function and the underlying regulatory mechanisms of the key m6A reader in NPC metastasis remain elusive.

Herein, we observed that the m6A reader IGF2BP3 was upregulated in NPC tissues and IGF2BP3 overexpression was an independent predictor of poor prognosis in NPC patients. Ectopically expressed IGF2BP3 expression in NPC cells identifies and binds with m6A-modified NOTCH3 mRNA to facilitate its stability and inhibit deadenylation-mediated mRNA decay, which constantly activates the Notch signaling pathway and enhances the tumor-initiating activity, eventually promoting tumor metastasis. In general, our findings shed light on the mechanism of tumor metastasis in NPC and suggest a novel therapeutic strategy against NPC tumor metastasis.

## Results

### IGF2BP3 is upregulated in NPC and associated with metastasis and poor prognosis

**Fig. 1:**
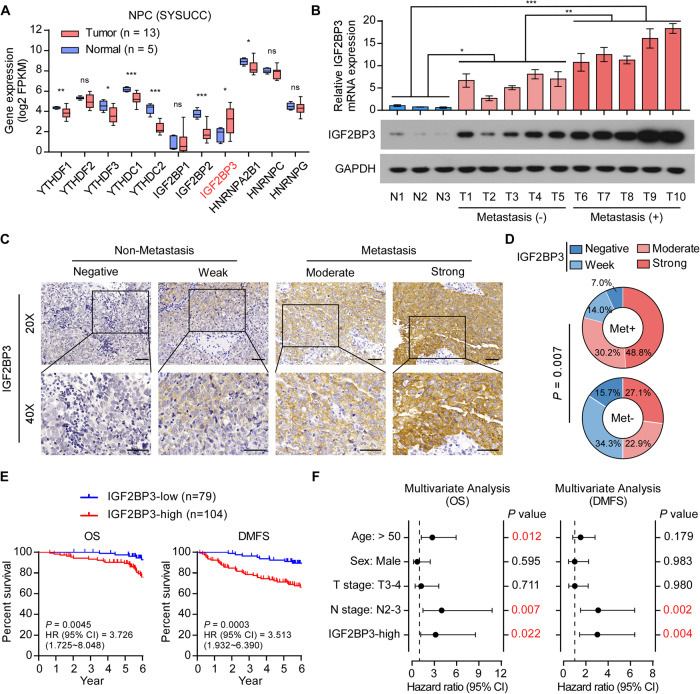
IGF2BP3 upregulation correlates with NPC metastasis. **A** The gene expression m6A reader genes in the RNA-seq analysis. **B** The mRNA (upper) and protein (lower) expression levels of IGF2BP3 in normal nasopharyngeal epithelial tissues, metastatic NPC and non-metastatic NPC. GAPDH was used as the loading control. Each error bar represents the mean ± SD of three independent experiments. **C** Representative IHC image of IGF2BP3 expression in patients with metastatic (*n* = 41) and non-metastatic (*n* = 142) NPC. Scale bars: 50 μm. **D** Quantification of IGF2BP3 IHC staining in patients with metastatic (*n* = 41) and non-metastatic (*n* = 142) NPC. **E** Kaplan–Meier analysis of overall survival (OS) and distant metastasis-free survival (DMFS) of patients with NPC (*n* = 183) grouped by low and high IGF2BP3 expression. **F** Multivariable Cox regression analysis to evaluate the significance of the association between high IGF2BP3 expression and DMFS or OS in the presence of other important clinical variables. *P < 0.05; **P < 0.01; ***P < 0.001; ns no significance.

The fate of m6A-modificated RNA is executed by m6A readers; therefore, we explored the key m6A readers in NPC. RNA-sequencing analysis was performed on 13 of NPC and 5 of normal nasopharyngeal epithelium samples. Focusing on these m6A readers, IGF2BP3 mRNA levels were significantly upregulated in NPC (Fig. 1A). Notably, we collected additional 13 frozen tissues to detect the mRNA and protein levels of IGF2BP3, in which the IGF2BP3 expression level were obviously increased in NPC tissues compared with those in normal tissues, especially in metastatic NPC tissues (Fig. 1B). The clinical significance of IGF2BP3 expression was assessed using immunohistochemistry (IHC) in 183 paraffin-embedded NPC specimens (Fig. 1C and Supplementary Table S1). 104 out of the 183 (56.8%) NPC patients had high IGF2BP3 expression, which presented a staining intensity ≥2, while 43.2% had low IGF2BP3 expression. Correlation analysis showed that high IGF2BP3 expression significantly correlated with NPC distant metastasis (Fig. 1D). Moreover, high IGF2BP3 expression was associated with advanced tumor-node-metastasis (TNM) stage (*P* = 0.037), advanced N stage (*P* = 0.005), tumor metastasis (*P* = 0.001) and patient death (*P* = 0.008) (Supplementary Table S2). The Kaplan–Meier survival curves revealed that high IGF2BP3 expression was associated with shorter overall survival (OS) and distant metastasis-free survival (DMFS) in patients with NPC (Fig. 1E). Multivariate Cox regression analyses showed that IGF2BP3 expression level was determined as an independent prognostic factor for OS and DMFS in patients with NPC (Fig. 1F). Consistently, the significance statistic of IGF2BP3 in multivariate analyses of OS disappeared when metastasis was added as a variable (Supplementary Fig. 1A). Collectively, these findings suggested that IGF2BP3 upregulation correlates with NPC metastasis.

### IGF2BP3 regulates tumor metastasis in NPC

**Fig. 2:**
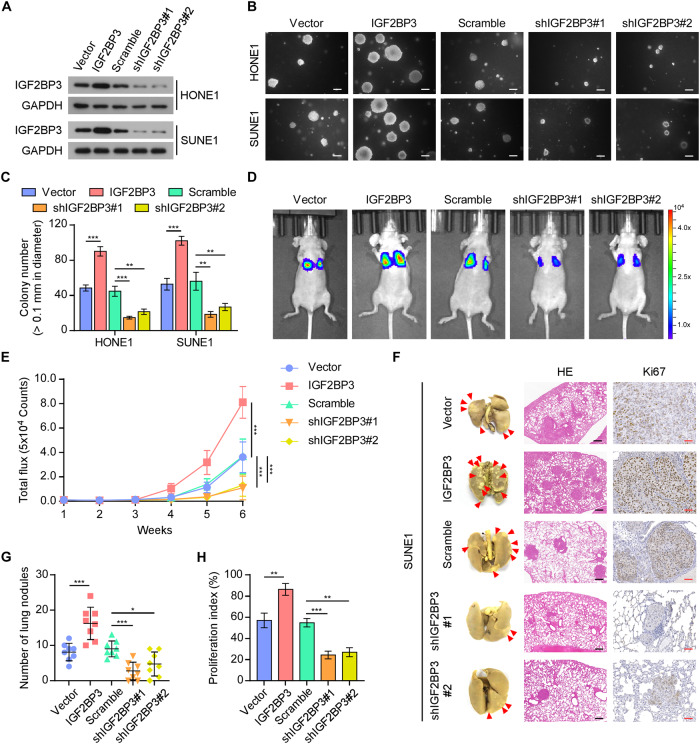
IGF2BP3 regulates NPC metastatic colonization ability. **A** The protein levels of IGF2BP3 in the indicated cells were detected by western blotting. GAPDH was used as the loading control. Representative images (**B**) and quantification (**C**) of anchorage-independent colony formation for the indicated cells; data are presented as the mean ± SD of three independent experiments. Scale bars: 200 µm. **D** Representative luciferase signal images of tumor-bearing mice at 6 weeks after inoculation. **E** The lung metastasis burden was evaluated by quantifying the luciferase signals every week. **F** Lung metastases of mice and representative H&E and Ki67 staining images are shown, as indicated by an arrow. Black scale bars: 250 µm; Red scale bars: 50 µm. **G** Quantitative analysis of visible surface metastatic lesions on the lung in each group. **H** Quantification of IHC analysis of Ki67 signals in the lung metastatic foci. Each error bar in (**E**) and (**G**, **H**) represent the mean ± SD of the tumor mouse models (*n* = 8/group). *P < 0.05; **P < 0.01; ***P < 0.001.

During the process of tumor metastasis, large numbers of cancer cells disseminate from the primary tumor, but only a small proportion of DTCs survive on infiltrating distant organs, which is a precondition for successful metastatic colony formation [6, 7]. To explore whether IGF2BP3 affects this inefficient process, we selected the HONE1 and SUNE1 cell lines, with a medium level of IGF2BP3 expression, to overexpress or knockdown IGF2BP3 (Supplementary Fig. 2A and Fig. 2A). Strikingly, upregulating IGF2BP3 significantly promoted, while silencing IGF2BP3 suppressed the anchorage-independent growth of NPC cells in vitro (Fig. 2B, C). Furthermore, we constructed a nude mouse xenograft model of lung metastasis. The indicated luciferase expressing cells (SUNE1-Vector, SUNE1-IGF2BP3, SUNE1-Scramble, SUNE1-shIGF2BP3#1, or SUNE1-shIGF2BP3#2) were intravenously injected into the tail veins of nude mice. Overexpression of IGF2BP3 significantly augmented, whereas silencing of IGF2BP3 reduced the bioluminescence intensity in the lung compared with that of the control group (Fig. 2D, E). After resection of the lungs at 6 weeks after injection, more tumor nodes were found on lung surfaces of mice in the IGF2BP3 overexpression group, compared with those in the control group. By contrast, IGF2BP3 silencing significantly decreased the number of disseminated tumor nodules in the lungs (Fig. 2F, G). Moreover, Ki67 staining confirmed that the tumors in the mice in the IGF2BP3 overexpression group had better proliferation potential (Fig. 2F–H). These findings indicated that IGF2BP3 promotes distant metastasis of NPC.

### IGF2BP3 promotes the cancer stemness of NPC

**Fig. 3:**
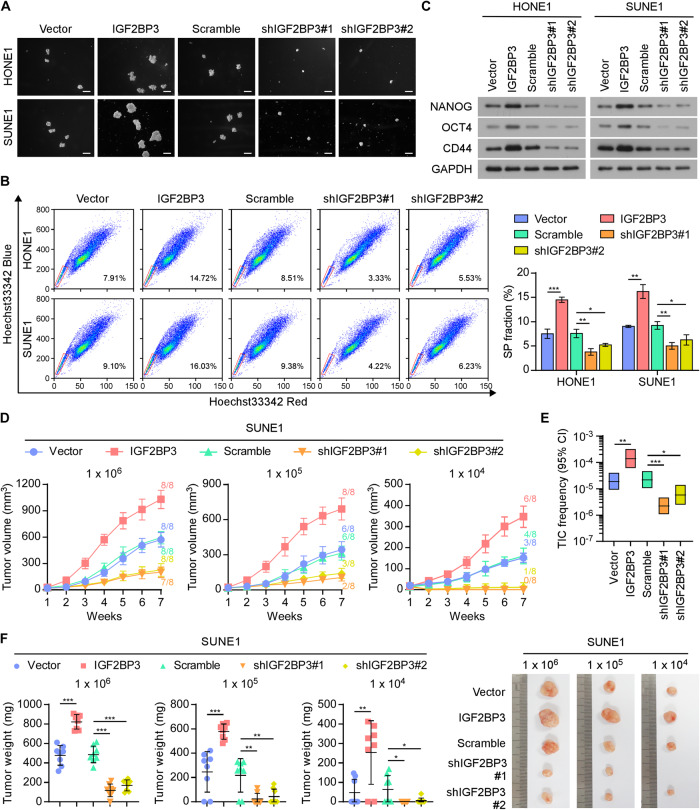
IGF2BP3 promotes cancer stemness of NPC. **A** Representative images of the tumor spheres formed in the indicated HONE1 and SUNE1 cells. Scale bars: 200 µm. **B** Representative images (left) and quantification (right) of the side-population cells in the indicated HONE1 and SUNE1 cells. **C** Western blotting analyses of the stemness markers (NANOG, OCT4, and CD44) in the indicated HONE1 and SUNE1 cells. GAPDH was used as the loading control. **D** Tumor volume curves of the different groups. **E** Frequency of tumor-initiating cells in the different groups analyzed using ELDA software (http://bioinf.wehi.edu.au/software/elda/). **F** Tumor weigh (left) and representative images of tumors (right) of the indicated groups. Each error bar in (**B**) represents the mean ± SD of three independent experiments. Each error bar in (**D**) and (**F**) represent the mean ± SD of the tumor mouse models (*n* = 8/group). *P < 0.05; **P < 0.01; ***P < 0.001.

The survival and tumor-initiating activity of DTCs have a fundamental role in metastatic colonization [8]. The sphere formation assay presented that IGF2BP3 overexpression resulted in the formation of significantly more and larger spheres compared with those in the vector control group. Contrastingly, IGF2BP3 silencing resulted in fewer and smaller spheres compared with those in the scramble group (Fig. 3A and Supplementary Fig. 3A). Moreover, the proportion of side-population (SP) cells in the IGF2BP3 overexpression group was increased, while it was reduced in the IGF2BP3 knockdown groups (Fig. 3B). Consistently, the levels of cell stemness markers, NANOG, OCT4, and CD44, were increased markedly by IGF2BP3 overexpression, but decreased in IGF2BP3-silenced cells (Fig. 3C). We further injected limiting numbers (1 × 10^6^, 1 × 10^5^, 1 × 10^4^) of the indicated cells into nude mice to evaluate the function of IGF2BP3 in regulating the cancer stem cell (CSC) properties of NPC in vivo. IGF2BP3 upregulation contributed to markedly higher tumor incidence and faster xenograft outgrowth. Conversely, the IGF2BP3-silenced cells presented lower tumor incidence and slower xenograft outgrowth (Fig. 3D–F). Consistently, IGF2BP3 overexpression increased the tumor initiating cell (TIC) frequency, whereas IGF2BP3 silencing had the reverse effect (Fig. 3E). Notably, ectopic expression of IGF2BP3 potently increased NANOG, OCT4, and CD44 levels in NPC tumors, while silencing IGF2BP3 potently impaired stemness marker levels in tumors (Supplementary Fig. 3B). These results indicated that IGF2BP3 plays a vital role in maintaining the cancer stemness in NPC.

### IGF2BP3 promoted the activation of Notch3 pathway via stabilizing NOTCH3 mRNA

**Fig. 4:**
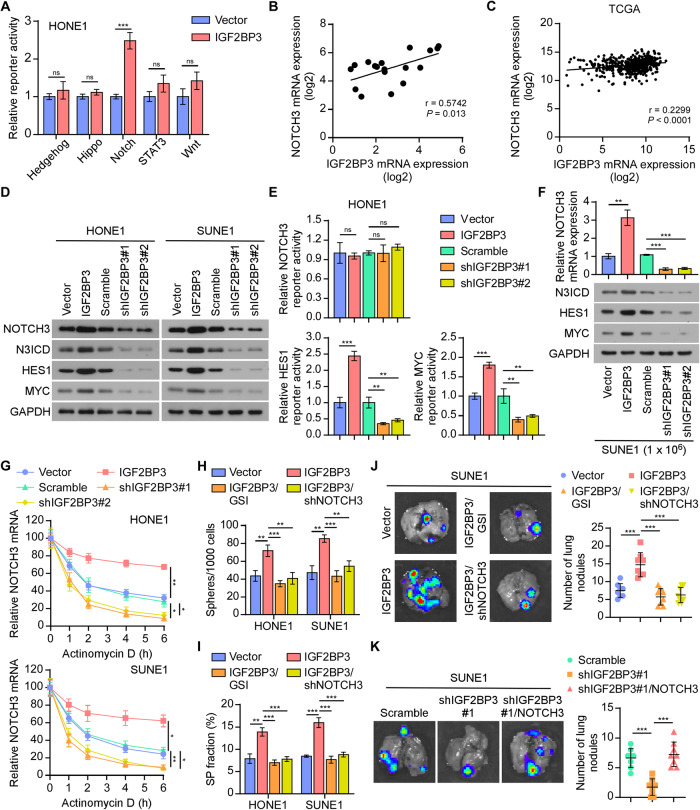
IGF2BP3 promoted the activation of Notch3 pathway via stabilizing NOTCH3 mRNA. **A** Luciferase reporter assays of Hedgehog, Hippo, Notch, STAT3, and Wnt/β-catenin reporters in HONE1 cells. **B** Correlation between IGF2BP3 and NOTCH3 mRNA expression in RNA-seq data. **C** Correlation between IGF2BP3 and NOTCH3 mRNA expression in the TCGA HNSC dataset. **D** Western blotting analyses the levels of critical proteins in the Notch3 signaling pathway (NOTCH3, N3ICD, HES1, and MYC) in the indicated groups. GAPDH was used as the loading control. **E** HES1 and MYC promoter activity was analyzed in the indicated HONE1 cells. **F** qRT-PCR of NOTCH3 (upper) and western blotting analysis of Notch3 signaling pathway (lower) in NPC tumors. GAPDH was used as the loading control. **G** The indicated NPC cells were treated with actinomycin D (5 µg/ mL). RNA was isolated at the specified time point and *NOTCH3* was subsequently analyzed by qRT-PCR analysis. The half-life of the mRNA was tracked by calculating its level relative to the untreated cells. **H** Quantification of sphere formation in the different cells. **I** Quantification of the side-population cells among the indicated cells. **J** Representative bioluminescence images (left) and quantification (right) of visible surface metastatic lesions on the lung in each group. **K** Representative bioluminescence images (left) and quantification (right) of visible surface metastatic lesions on the lung in indicated group. Data in (**J**) and (**K**) are presented as the mean ± SD of tumor mouse models (*n* = 8/group). Each error bar in (**A**), (**E**), and (**G**–**I**) represent the mean ± SD of three independent experiments. *P < 0.05; **P < 0.01; ***P < 0.001.

To determine the molecular mechanism underlying IGF2BP3’s effects on CSC properties and NPC metastasis, we detected the activity of several classical stemness-related signaling pathways, including Hedgehog, Hippo, Notch, STAT3, and Wnt/β-catenin pathways. IGF2BP3 overexpression in HONE1 and SUNE1 cells dramatically increased the transcriptional activity of the Notch signaling pathway, but caused no significant change in the other signaling pathways (Fig. 4A and Supplementary Fig. 4A). Canonical Notch signaling is initiated by receptor-ligand interactions between neighboring cells, and then the active Notch intracellular domains (NICDs) of the receptors are released from the plasma membrane and translocated into the nucleus. Subsequently, the NICDs activate the transcription of target genes, including the HES family and MYC, which has been known to contribute to tumor stemness and metastasis [9, 14, 29]. We analyzed the RNA-seq data and found a significantly positive correlation between the mRNA levels of IGF2BP3 and NOTCH3, but not other Notch family members (Fig. 4B and Supplementary Fig. 4B). This positive correlation was also demonstrated in TCGA HNSC datasets (Fig. 4C). Moreover, the Notch3 signaling pathway was highly activated in NPC cell lines compared with that in the normal nasopharyngeal epithelial cell line (Supplementary Fig. 4C). These results suggest a biological regulation between IGF2BP3 and Notch signaling. Furthermore, IGF2BP3 upregulation dramatically promoted, while IGF2BP3 knockdown greatly reduced the mRNA and protein levels of NOTCH3 without affecting its transcription (Fig. 4D, E and Supplementary Fig. 4D, E). Consistent with this, IGF2BP3 overexpression increased the expression of Notch signaling target genes HES1 and MYC (Fig. 4D). Luciferase reporter assays showed that IGF2BP3 overexpression promoted, whereas IGF2BP3 silencing dramatically weakened the luciferase activity of the HES1 and MYC promoters (Fig. 4E and Supplementary Fig. 4E). Likewise, ectopic expression of IGF2BP3 potently promoted, while IGF2BP3 downregulation impaired the mRNA levels of NOTCH3 and the Notch3 signaling activation in tumors (Fig. 4F).

We next investigated the stability of NOTCH3 mRNA by different time of Actinomycin D treatment. Knockdown of IGF2BP3 significantly decreased, while overexpression of IGF2BP3 dramatically increased the stability and half-life of NOTCH3 mRNA (Fig. 4G). In addition, we used RO4929097, a γ-secretase inhibitor (GSI), or NOTCH3-targeted shRNAs to inhibit Notch3 pathway in IGF2BP3-overexpressing cells and revealed that suppressing the Notch3 pathway abrogated the role of IGF2BP3 in promoting HES1 and MYC expression (Supplementary Fig. 4F, G). Furthermore, GSI treatment and silencing NOTCH3 significantly abrogated IGF2BP3-mediated tumor-initiating ability (Fig. 4H, I). Inhibition of NOTCH3 resulted in a significant reduction in metastasis of NPC cells induced by IGF2BP3 (Fig. 4J). Notably, silencing IGF2BP3 potently impaired the stemness properties in NPC cell lines and lung metastasis in tumors, and these effects were abrogated by NOTCH3 overexpression, suggesting that IGF2BP3 promotes tumor metastasis through Notch3 pathway (Supplementary Fig. 4H, I and Fig. 4K). These results presented that IGF2BP3 stabilized NOTCH3 mRNA and sustained Notch3 pathway activation to promote NPC tumor metastasis.

### IGF2BP3 facilitates NOTCH3 mRNA stability in an m6A-dependent manner

**Fig. 5:**
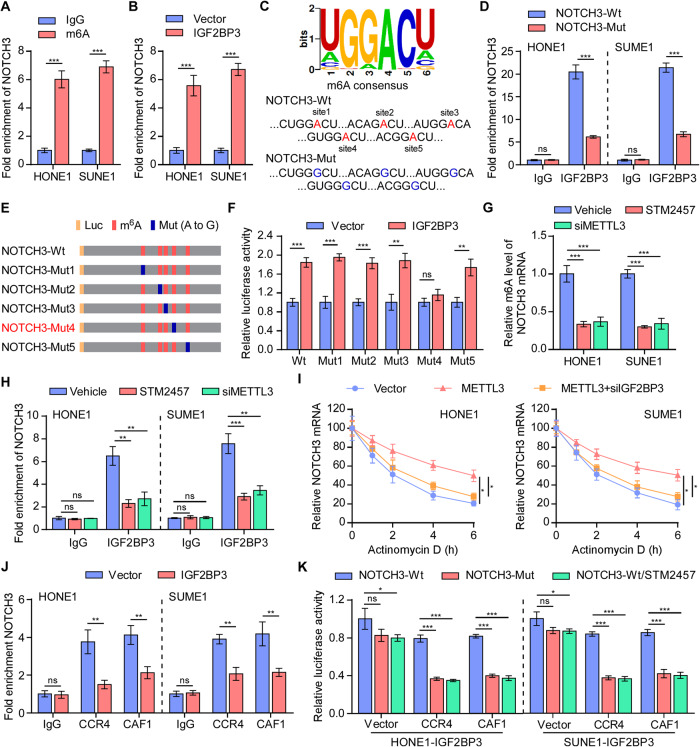
IGF2BP3 facilitates NOTCH3 mRNA stability in an m6A-dependent manner. **A** M6A RIP-qPCR assay analysis of the levels of m6A-modificated *NOTCH3* in HONE1 and SUNE1 cells. **B** Endogenous RIP assays followed by qRT-PCR analysis examines the interaction between IGF2BP3 and *NOTCH3* mRNA. **C** Schematic illustration of the putative wild-type m6A sites and designed mutant m6A sites in the NOTCH3 transcript. **D** RIP-qPCR assays showing the enrichment of *NOTCH3* on IgG and IGF2BP3 in the NOTCH3-Wt or NOTCH3-Mut NPC cells. **E** Schematic illustration of mutated (GGAC to GGGC) sites in *NOTCH3*. **F** The luciferase activities of different mutated NOTCH3 reporters in the indicated groups. **G** Relative m6A level of *NOTCH3* in NPC cells with or without METTL3 silencing or METTL3 inhibition. **H** RIP-qPCR assays show the enrichment of NOTCH3 mRNA on IgG and IGF2BP3 in NPC cells with or without METTL3 silencing or METTL3 inhibition. **I** The indicated SUNE1 cells were treated with actinomycin D (5 µg/ mL). RNA was isolated at the specified time point and *NOTCH3* was subsequently analyzed by qRT-PCR analysis. The half-life of the mRNA was tracked by calculating its level relative to that in the untreated cell. **J** RIP assays followed by qRT-PCR to examine the interaction between CCR4 or CAF1 and NOTCH3 mRNAs in indicated NPC cells. **K** The luciferase activities of NOTCH3-Wt upon CCR4 or CAF1 overexpression compared to NOTCH3-Mut reporters or the addition of the METTL3 inhibitor in IGF2BP3-overexpressing NPC cells. Each error bar represents the mean ± SD of three independent experiments. *P < 0.05; **P < 0.01; ***P < 0.001; ns no significance.

It is uncertain whether NOTCH3 mRNA regulated by m6A modifications, which has been identified to affect mRNA stability [22, 26]. Subsequently, we found that the NOTCH3 mRNA sequence was enriched in m6A modifications in NPC cells (Fig. 5A). Endogenous RNA immunoprecipitation (RIP) assays performed in NPC cells confirmed that IGF2BP3 consistently interacted with NOTCH3 transcripts (Fig. 5B). However, either upregulation or silencing of IGF2BP3 had no effects on m6A modifications levels of NOTCH3 mRNA (Supplementary Fig. 5A). As an m6A readers, IGF2BP3 targets thousands of mRNA transcripts through the recognition of m6A motifs [26]. Therefore, we explored whether IGF2BP3 directly bound to NOTCH3 mRNA depending on its m6A modification. Analysis of the public MeRIP-Seq data of NPC tissues in PRJCA004279 showed that the m6A modifications of NOTCH3 were predominately located in the 3′ untranslated region (UTR) and coding regions (CDS) (Supplementary Fig. 5B) [30]. The online RNA modification website (SRAMP, http://www.cuilab.cn/sramp) predicted five high confidence m6A sites within the NOTCH3 CDS (Supplementary Fig. 5C). In consistent with this, the region encompassing the five putative m6A sites (3602 ~ 5593nt) was indispensable for the interaction with IGF2BP3 protein (Supplementary Fig. 5D). Similarly, the direct binding between NOTCH3 mRNA and IGF2BP3 was impaired by mutation of all putative m6A sites (Fig. 5C, D). Furthermore, to investigate which m6A site is responsible for m6A‐mediated *NOTCH3* stabilization, five NOTCH3 mutants were designed and dual-luciferase reporter assays were performed (Fig. 5E). Overexpression of IGF2BP3 promoted the luciferase expression of all NOTCH3 mutants, except for NOTCH3-mut4, indicating that site 4 (‘GUGGACU’) is involved in regulating *NOTCH3* stability (Fig. 5F).

Silencing of METTL3 had the most significant effects on the reduction of *NOTCH3* expression in NPC cell (Supplementary Fig. 5E). It has been reported that METTL3 catalyzes the m6A modification of Notch receptor family mRNAs [16, 31]. STM2457 is a potent and selective inhibitor of METTL3 catalytic activity [32]. In line with this, we found that using the specific siRNAs or inhibitor of METTL3 decreased the m6A modification of *NOTCH3*, eventually reduced the NOTCH3 expression in NPC cells (Supplementary Fig. 5F and Fig. 5G). Further, the results of RIP assays suggested that depletion of METTL3 dramatically suppressed *NOTCH3* enrichment on IGF2BP3 (Fig. 5H); and the mRNA stability assay demonstrated that depletion of IGF2BP3 significantly impaired the half-life of NOTCH3 mRNA even when *NOTCH3* had a high level of m6A-modification (Fig. 5I). Overall, these findings indicated that IGF2BP3 promotes NOTCH3 mRNA stability in a m6A-dependent manner.

Deadenylation-dependent mRNA turnover is the primary pathway for the degradation of most mRNAs in eukaryotes, in which the CCR4-NOT complex mediates the shortening of poly(A) tails [33, 34]. To investigate whether the IGF2BP3-mediated increase in *NOTCH3* is related to CCR4-NOT complex-mediated deadenylation, we conducted exogenous RIP assays. The results indicated that overexpressing IGF2BP3 dramatically impaired the binding between *NOTCH3* and deadenylases CCR4 or CCR4-associated factor 1 (CAF1) (Fig. 5J). Further analysis revealed that either upregulating or silencing IGF2BP3 had no effect on CCR4 and CAF1 expression (Supplementary Fig. 5G). Moreover, CCR4 and CAF1 overexpression slightly accelerated the deadenylation of the NOTCH3-wt reporter in IGF2BP3-overexpressed cells, whereas such acceleration was augmented in the NOTCH3-mut reporter or by the addition of the METTL3-specific inhibitor, suggesting that the m6A modification could protect *NOTCH3* from CCR4-NOT complex-mediated deadenylation (Supplementary Fig. 5H and Fig. 5K). Taken together, these results implied that IGF2BP3 promoted NOTCH3 mRNA stability by suppressing CCR4-NOT complex-mediated deadenylation in an m6A-dependent manner.

### Clinical relevance and study model

**Fig. 6:**
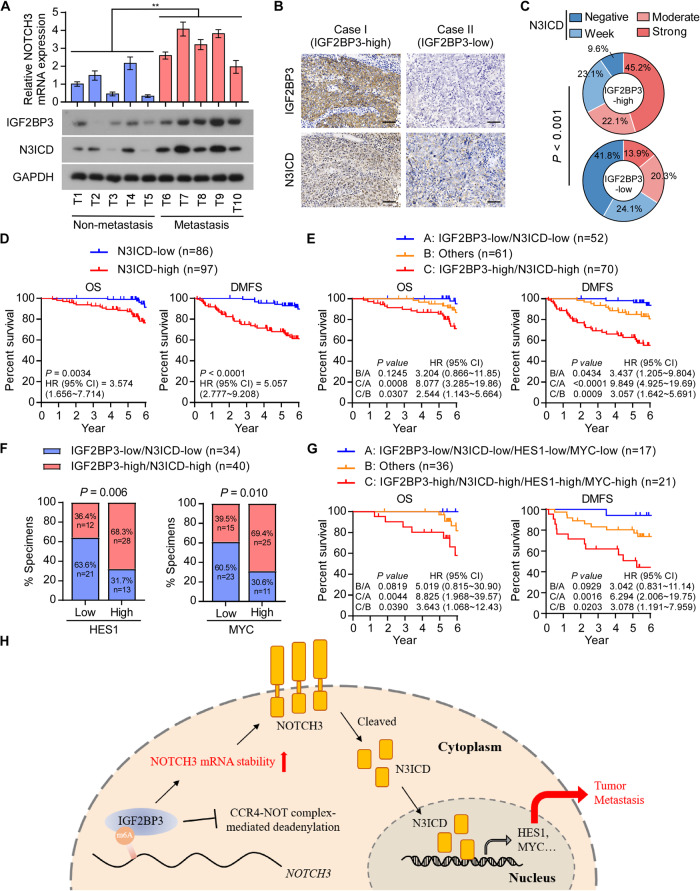
Clinical relevance and study model. **A** qRT-PCR of *NOTCH3* (upper) and western blotting analysis of IGF2BP3 and N3ICD (lower) in primary NPC tissue with or without metastasis, GAPDH was used as the loading control. **B** Representative images of IGF2BP3 and N3ICD IHC staining in specimens from patients with NPC (*n* = 183). Scale bars: 50 μm. **C** Correlation analysis showed that IGF2BP3 was significantly associated with the expression scores of N3ICD. **D** Kaplan–Meier analysis of OS and DMFS of patients with NPC grouped by the expression scores of N3ICD. **E** Kaplan–Meier analysis of OS and DMFS of patients with NPC divided into three groups, including IGF2BP3-high/N3ICD-high, IGF2BP3-low/N3ICD-low and others. **F** Correlation analysis revealed that the mRNA levels of HES1 and MYC were significantly associated with co-expression of IGF2BP3/N3ICD in patient specimens (*n* = 74). **G** Kaplan–Meier analysis of OS and DMFS of patients with NPC divided into indicated groups. **H** Study model: IGF2BP3 facilitates NOTCH3 mRNA stability to sustain the activation of the Notch3 pathway and promote tumor metastasis in NPC. **P < 0.01.

Finally, we examined whether the IGF2BP3/Notch3 axis was clinically relevant in NPC. IGF2BP3 and N3ICD levels were potently upregulated in NPC primary tumor samples from the patients with metastatic NPC compared to those without metastasis, suggesting the significant relevance of the IGF2BP3/Notch3 axis in NPC (Fig. 6A). Furthermore, we evaluated N3ICD and IGF2BP3 levels by IHC staining in paraffin-embedded NPC samples. The IHC staining and correlation analysis showed that IGF2BP3 positively correlated with N3ICD levels in the clinical samples (Fig. 6B, C). Similar to IGF2BP3, high N3ICD levels correlated with shorter OS and DMFS in patients with NPC (Fig. 6D). Importantly, patients with combined high IGF2BP3 and high N3ICD levels suffered the worse OS and DMFS (Fig. 6E). In addition, we detected the mRNA levels of HES1 and MYC in 74 out of the 122 (60.7%) NPC tissues with low or high co-expression of IGF2BP3 and N3ICD. Correlation analysis showed that high mRNA levels of HES1 and MYC significantly correlated with high co-expression of IGF2BP3 and N3ICD (Fig. 6F). Moreover, patients in high co-expression of IGF2BP3 and N3ICD combined with high mRNA levels of HES1 and MYC exhibited the shorter OS and DMFS (Fig. 6G), further suggesting that the IGF2BP3/Notch3 axis was indeed associated with poor clinical outcomes of NPC.

In summary, we here show that IGF2BP3 facilitates NOTCH3 mRNA stability to promote Notch3 signaling activation, then enhances the survival and tumor-initiating activity in disseminated tumor cells, leading to robust tumor metastasis in NPC (Fig. 6H). This study uncovers a mechanism for tumor metastasis in NPC, suggesting that inhibition of IGF2BP3 might be a promising strategy.

## Discussion

A high potential for distant metastasis results in the majority of treatment failure and deaths in patients with NPC [3–5]. Therefore, there is an urgent need to identify the pivotal molecules that drive NPC metastasis and develop promising targeted therapies to improve the clinical outcome. Herein, we revealed that IGF2BP3 was distinctly upregulated in NPC tissues, especially in those with distant metastasis, and predicted unfavorable outcomes in patients. Mechanistically, IGF2BP3 activates Notch3 signaling by maintaining NOTCH3 mRNA stability in an m6A-dependent manner, thus enhancing the tumor-initiating ability of DTCs and promoting the development of metastasis. Our findings highlight a novel mechanism of constant Notch3 signaling activation in NPC metastasis, and suggest IGF2BP3 as a potent prognostic biomarker and a potential target against progressive metastasis in NPC.

As an evolutionarily conserved mechanism, the Notch3 pathway is one of a major signaling axis in the maintenance of the stem-like phenotype, in which the functions of Notch signaling are not limited to triggering tumorigenesis and enhancing drug resistance, but instead exert multiple pivotal functions in tumor metastasis [9, 35–39]. For example, matrix metalloproteinase 28 (MMP28) enhances epithelial-mesenchymal transition (EMT) and promotes hepatocellular carcinoma metastasis via activating Notch3 Signaling [37]. *MUC4/Y*, one of the MUC4 transcript variants, was reported to be overexpressed in pancreatic cancer, in which it upregulated NOTCH3 expression, promoting tumor angiogenesis and metastasis [38]. In the current study, we demonstrated that Notch3 signaling plays a fundamental role in the IGF2BP3-induced stem-like phenotype of NPC cells, which eventually leads to distant metastasis. Therefore, targeting Notch3 signaling appears to be a rational and innovative approach for the treatment of NPC.

GSIs are a major class of Notch inhibitors, which prevent the final proteolytic cleavage of Notch receptors that releases the NICD [14]. It has been verified that has significant antitumor effects in preclinical studies of advanced solid tumors [40–42]. Currently, only preclinical studies have been performed in NPC, which revealed that GSIs inhibit proliferation and enhances the radiosensitivity of NPC cells [17, 18]. Consistently, our study demonstrated that a GSI reduced the tumor initiating ability of NOTCH3-upregulated NPC cells and effectively suppressed NOTCH3-mediated lung metastasis in vivo. Nevertheless, phase II trials of metastatic pancreatic and colorectal cancer showed that GSI had only minimal clinical activity and the clinical outcomes were not superior to historical treatment data in unselected populations [43, 44]. Based on the importance of Notch3 signaling in metastatic NPC, we anticipated there will be more applications for GSIs and other agents that target Notch signaling to limit metastasis. It is further need of selecting optimal patients in NPC to received targeting Notch signaling agents, according to prescreening for the presence of Notch3 signaling activation.

Besides post-translational regulation, given that adenine/uridine-rich elements (AREs) occur in the 3′ UTR of Notch-class genes in humans, modulation of RNA metabolism is likely to be involved in Notch signaling regulation [45]. Indeed, ZFP36L1 and ZFP36L2 suppressed NOTCH1 expression in T cell acute lymphoblastic leukemia by interacting with the 3′ UTR of *NOTCH1* [46]. YTHDF2, an m6A binding protein, acts as an intrinsic suppressor in Notch signaling by promoting NOTCH1 mRNA degradation under normal cellular conditions [13]. However, post-transcriptional regulation of NOTCH3 in NPC remains to be further explored. Commonly, the mRNA decay directed by AREs in the 3′ UTR are triggered by deadenylation [47]. In our study, we demonstrated that IGF2BP3 binds with m6A-modified *NOTCH3* and promotes its mRNA stability via decreasing the interaction between *NOTCH3* and the CCR4-NOT deadenylase complex, leading to constantly activated Notch3 signaling. Our findings reveal a novel post-transcriptional mechanism for the activation of Notch3 signaling.

IGF2BP3, recently identified as a novel m6A reader, has been found elevated in various cancers and involved in the tumorigenesis and tumor progression [27]. Previous evidence suggested that IGF2BP3 was upregulated in NPC tissues and promoted EMT by activating AKT/mTOR signaling [48], which could be activated in the noncanonical Notch signaling pathway [14, 49]. Besides, we found that IGF2BP3 was the most significantly upregulated m6A reader in metastatic NPC and predicted unfavorable clinical outcomes. IGF2BP3 augmented Notch3 signaling via maintaining NOTCH3 mRNA stability in a m6A-dependent manner, ultimately promoting NPC metastasis. In addition, Du et al. revealed that MYC-activated IGF2BP3 promoted NPC cell proliferation and metastasis [50]. MYC is regarded as a downstream targets of Notch signaling pathway [51], which was proven to be upregulated in IGF2BP3-mediated Notch3 signaling overactivation. However, whether the IGF2BP3/Notch3/MYC axis forms a positive feedback loop to regulate NPC metastasis requires further research. Hence, IGF2BP3 plays a critical role in regulating NPC metastasis. Intriguingly, IGF2BP3 was regarded as an RNA-binding proteins (RBPs), for which several clinical trials have emerged recently to evaluate the anti-tumor efficacy of specific antisense oligonucleotide (ASO) [52, 53]. This RNA interference-based approach could modulate the expression and activity of RBPs to regulate cancer-related pathway activation, which might shed light on novel therapeutic avenues for NPC treatment.

To sum up, our study revealed that IGF2BP3 acts as an oncogene to promote NPC metastasis by maintaining NOTCH3 mRNA stability via suppressing CCR4-NOT complex-mediated deadenylation, thus leading to constant activation of the Notch3 signaling pathway and enhancing tumor metastasis. Collectively, our findings highlighted an important role of IGF2BP3 in NPC metastasis and elucidated the precise mechanism of IGF2BP3-mediated post-transcriptional regulation on NOTCH3, identifying IGF2BP3 as a novel prognostic biomarker and potential therapeutic target in NPC metastasis.

## Materials and methods

### Cell lines and cell culture

The State Key Laboratory of Oncology in South China (Sun Yat-sen University Cancer Center, Guangzhou, China) provided the NP69 cell line (normal nasopharyngeal epithelial cells) and all the NPC cell lines. The NP69 cells were cultured in keratinocyte serum‐free medium supplemented with bovine pituitary extract. NPC cell lines, including C666-1, HNE1, HONE1, HK1, and SUNE1, were grown in RPMI 1640 (Invitrogen, Carlsbad, CA, USA) medium supplemented with 10% FBS (Life Technologies, Carlsbad, CA, USA) and 1% penicillin/streptomycin (Invitrogen, Carlsbad, CA, USA). All cell lines were maintained at 37 °C with 5% CO_2_. All cell lines were checked for no mycoplasma contamination and authenticated using short tandem repeat (STR) profiling.

### Patients and tissue samples

We collected 183 paraffin-embedded samples from patients who were diagnosed clinically and pathologically NPC between 2012 and 2016 at the Sun Yat-Sen University Cancer Center, and subjected them to immunohistochemistry analysis. The clinical information for these samples is shown in Supplementary Table S1. Thirteen frozen tissues (3 normal nasopharyngeal epithelial tissue and 10 NPC tumor tissues) were used for qRT-PCR and western blotting analysis. TNM classification of NPC was based on the Union for International Cancer Control and American Joint Commission on Cancer (8th edition, 2017). Tumor recurrence was defined as local relapse after radical treatment during 6-year follow-up. Tumor metastasis was defined as any type of distant metastasis after radical treatment at follow-up evaluations, including lung, bone, or liver metastasis. Disease progression included tumor recurrence and metastasis. All patients provided written informed consent, and the study was approved by the Internal Review and Ethics Board of Sun Yat-sen University Cancer Center (Approval No.GZR2020-148).

### RNA extraction, reverse transcription, and quantitative real-time PCR

Total RNA was isolated from cells using Trizol (Invitrogen, Carlsbad, CA, USA). Total RNA (1 ug) was reverse transcribed into cDNA using a GoScript Reverse Transcription System (Promega, Madison, WI, USA) in a 20 μl reaction mixture. cDNA was detected using a Bio-Rad CFX qRT-qPCR detection system (Bio-Rad, Hercules, CA, USA), and quantified using SYBR Green Master Mix (ROX; Roche, Toronto, Canada). All data were normalized to the expression of housekeeping gene GAPDH.

### IHC

One hundred and eighty-three NPC tissues fixed in 10% formalin and embedded in paraffin were cut into 4 μm sections and placed on slides. The slides were incubated with anti-IGF2BP3 (ab177477, Abcam) and anti-NOTCH3 antibodies (Sc-5593, Santa Cruz Biotechnology) at 4 °C overnight. The IHC staining results were evaluated and scored by two independent pathologists who were blinded to the clinical outcome. The staining intensity was graded as strong +3, moderate +2, weak +1, and negative 0. Specimens with scores +3 and +2 were defined as high expression, while those that scored as +1 or 0 were defined as low expression. Staining of N3ICD was defined as nuclear NOTCH3 expression in the specimens.

### Xenograft tumor model

Male BALB/c-nude mice (4–5 weeks old) were housed in barrier facilities on a 12-h light/dark cycle and randomly divided into the indicated groups (8 mice/group). All animal experiments were approved by the Ethics Committee of Sun Yat-sen University Cancer Center (L102042021000K). For the lung metastasis model, SUNE1 cells (1 × 10^6^) were injected intravenously into the tail veins of nude mice. The growth of metastatic foci was detected using an in vivo imaging system (IVIS). The mice were sacrificed after 6 weeks and their lungs were removed. Samples were paraffin-embedded and subjected to H&E and IHC staining.

For the limiting dilution assays, 1 × 10^6^, 1 × 10^5^, or 1 × 10^4^ SUNE1 cells were injected subcutaneously into mice. Following sacrifice of the mice at after 7 weeks post-inoculation, tumors were excised, and the tumor volume and weight were analyzed. The frequency of tumor-initiating cells was calculated using the ELDA software (http://bioinf.wehi.edu.au/software/elda/).

No sample size calculations were performed. Sample size was determined according to our experience as well as literature reporting in terms of specific experiment. Randomization method was used to determine how animals were allocated to different groups. To achieve randomization, all animals were numbered by body weight, then, random number table was used to allocate animals to experimental groups. During the study, no data was excluded from the experiments, and no blinding was done.

### RNA stability assay

NPC cells were cultured in 6-well plates overnight, and then treated with 5 µg/ml actinomycin D (MedChemExpress) to inhibit gene transcription for various times. Next, RNA was extracted and analyzed using qRT‐PCR (normalized to GAPDH expression).

### RIP assays

The RIP assay was carried out using a Magna RIP RNA-Binding Protein Immunoprecipitation Kit (Millipore, Burlington, MA, USA), according to the manufacturer’s instructions. 1 × 10^7^ cells were required for each RIP procedure. In brief, NPC cells were lysed in complete RIP lysis buffer containing magnetic beads conjugated with anti-m6A (202003, Synaptic Systems), anti-IGF2BP3 (ab177477, Abcam), anti-CCR4 (ab221151, Abcam) or anti-CAF1 (ab195587, Abcam) at 4 °C overnight. The proteins were digested with proteinase K after the magnetic beads were washed. The purified RNA was used for qRT-PCR analysis.

### RNA sequencing data

RNA extraction and RNA sequencing were performed on 5 of normal nasopharynx and 13 of NPC tissues (one of them was a patient-derived xenograft). Differential expression analysis was performed using the DESeq2 software package, and all gene expression values were log2 transformed. The RNA-seq data have been deposited in the Genome Sequence Archive of the BIG Data Center at the Beijing Institute of Genomics, Chinese Academy of Science, under accession number HRA004724 (accessible at http://bigd.big.ac.cn/gsa-human).

### Statistical analysis

SPSS version 20.0 (SPSS Inc, Chicago, IL) and GraphPad Prism 7 version (GraphPad Software, La Jolla, CA, USA) were used for the statistical analyses. Log-rank, Chi-squared, One-way ANOVA, and Student’s two-tailed *t*-tests were used for data analysis. Multivariate statistical analysis was performed using a Cox regression model. *P* < 0. 05 was considered statistically significant.

More materials and methods are included in the Supplementary files (Supplementary information).

### Supplementary information

Supplementary information

Uncropped gel images

### Source data

Source data

## Data Availability

The authors confirm that the data supporting the findings of this study are available within the article and its supplementary materials, or available from the corresponding author on reasonable request.
