# Supplementary material for: The m6A reader IGF2BP3 preserves NOTCH3 mRNA stability to sustain Notch3 signaling and promote tumor metastasis in nasopharyngeal carcinoma
Source: Oncogene. 2023 Oct 18;42(48):3564–74. doi: 10.1038/s41388-023-02865-6 (PMC10673713; doi:10.1038/s41388-023-02865-6)
Supplement: Supplementary file 1 — Supplementary information [file 41388_2023_2865_MOESM1_ESM.pdf]

SUPPLEMENTARY INFORMATION

Supplementary Materials and Methods

Construction of plasmids and stable cell lines

Human cDNAs were PCR-amplified and cloned into the pLVX-IRES-puro vector for overexpression. The shRNAs against IGF2BP3 or NOTCH3 was cloned into the pSuper-retro-neo vector. Briefly, transfection of plasmids was performed using Lipofectamine 3000 reagent (Invitrogen) based on the manufacturer's instructions. Cells (2×10^5) were cultured and infected using a virus produced by the indicated plasmids for 3 days. Stable cell lines were selected using 0.5 μg/mL puromycin or 100μg/mL G418 for 7 days. The transfection efficiency was determined by RT-qPCR and western blotting assays. The m6A site - directed mutagenesis tagged with Flag was performed by gene synthesis, and the segment was integrated into the pcDNA3.1+ plasmid, which was confirmed by Sanger sequencing.

Western blotting analysis

Total proteins were obtained using sample buffer containing phosphatase Inhibitor Cocktail (CST, Danvers, MA, USA). Protein (30 μg) were separated by 9% SDS-PAGE and transferred onto PVDF membranes. After blocked with 5% skim milk, the PVDF membranes were incubated overnight at 4 °C with the primary antibodies, and then with the secondary antibodies. Immunoreactive proteins on the membranes were visualized using enhanced chemiluminescence reagents. Source Data are provided in a supplemental file. The antibodies used in this study were as follows: anti-IGF2BP3 (ab177477, dilution 1:1000; Abcam), anti-NOTCH3 (#5276, dilution 1:1000; CST), anti-HES1 (sc-166410, dilution 1:1000; Santa Cruz Biotechnology), anti-MYC (10828-1-AP, dilution 1:2000, Proteintech), anti-NANOG (#4903, dilution 1:2000, CST), anti-OCT4 (#75463, dilution 1:1000, CST), anti-CD44 (#37259, dilution 1:1000, CST), anti-METTL3 (#86132, dilution 1:1000, CST), and anti-GAPDH (#5174, dilution 1:1000; CST).

Anchorage-independent growth ability assay

One milliliter of complete medium with 1.3% agarose (Sigma-Aldrich) was plated as the bottom agarose layer, and NPC cells (n = 3000) digested using trypsin and suspended in 1 ml of complete

medium with 0.6% agarose were plated as the top layer in 6-well plates. Colonies larger than 100 μm were counted after 3 weeks of growth. The experiment was conducted three times independently.

Tumor sphere formation assays

NPC cells (n = 1000) were seeded in 6-well ultra-low cluster plates and cultured with DMEM/F12 serum-free medium (Invitrogen) supplemented with 5 μg/ml insulin (Sigma-Aldrich), 2% B-27 Supplement (Invitrogen), 0.4% bovine serum albumin (Sigma-Aldrich), 20 ng/ml basal fibroblast growth factor (PeproTech), and 20 ng/ml epidermal growth factor (PeproTech) for 14 days at 37 °C with 5% CO_2. All formed spheres were photographed using a ZEISS Vert. A1 microscope (Carl Zeiss, Jena, Germany) and spheres larger than 50 μm in diameter were counted using Image-Pro Plus 6.0 (Media Cybernetics Inc., Rockville, MD, USA).

Side population (SP) cell fraction assays

The indicated cells were resuspended in DMEM medium (ATCC-30-2003) containing 2% FBS (Gibco, Grand Island, NY, USA) at 1×10^6 cells per ml. Hoechst 33342 dye (Sigma-Aldrich) was added at a final concentration of 5 pg/ml in the presence or absence of verapamil (Sigma-Aldrich) and the cells were incubated at 37°C for 45 min with intermittent shaking. Control samples were treated with 100 μM verapamil (Sigma-Aldrich) to block dye efflux. At the end of the incubation, the cells were washed with cold PBS and subjected to flow cytometry analysis. The data were analyzed using Summit 5.2 software (Beckman Coulter, Indianapolis, IN, USA).

Luciferase activity assays

Cells (1×10^4) were grown in 48-well plates in triplicate for 24 h. Then, using Lipofectamine 3000 reagent (Invitrogen), 100 ng of the indicated plasmid or luciferase reporter plasmids, together with 1 ng of pRL-TK renilla plasmid (Promega), were transfected into the cells following the manufacturer's instructions. A Dual Luciferase Reporter Assay Kit (Promega) was then used to measure the activity signals of the renilla and luciferase enzymes.

Primers for qRT-PCR

IGF2BP3, forward primer, 5'-TATATCGGAAACCTCAGCGAGA-3';

reverse primer, 5'-GGACCGAGTGCTCAACTTCT -3';

NOTCH3#1, forward primer, 5'-TACTGGTAGCCACTGTGAGCAG-3';

reverse primer, 5'-CAGTTATCACCATTGTAGCCAGG-3';

NOTCH3#2, forward primer, 5'-CGTGGCTTCTTTCTACTGTGC-3';

reverse primer, 5'-CGTTCACCGGATTTGTGTCAC-3';

NOTCH3#3, forward primer, 5'-ATGCAGGATAGCAAGGAGGA-3';

reverse primer, 5'-AAGTGGTCCAACAGCAGCTT-3'

HES1, forward primer, 5'-ACGTGCGAGGGCGTTAATAC-3';

reverse primer, 5'-GGGGTAGGTCATGGCATTGA-3'

MYC, forward primer, 5'-GGCTCCTGGCAAAAGGTCA-3';

reverse primer, 5'-CTGCGTAGTTGTGCTGATGT-3'

GAPDH, forward primer, 5'-GTCTCCTCTGACTTCAACAGCG-3';

reverse primer, 5'-ACCACCCTGTTGCTGTAGCCAA-3';

ACTB, forward primer, 5'-CACCATTGGCAATGAGCGGTTC-3';

reverse primer, 5'- AGGTCTTTGCGGATGTCCACGT-3'.

Gene silencing oligonucleotides

shIGF2BP3#1, target sequence: 5'-GCACCTCTGCGGCTTGTAAGT-3'

shIGF2BP3#2, target sequence: 5'-GCTGGTTCCCACCCAATTTGT-3'

shNOTCH3, target sequence: 5'-GCATGAAGAACATGGCCAAGG-3'

siMETTL3, target sequence: 5'-GCACTTGGATCTACGGAAT-3'5'--3'

siMETTL14, target sequence: 5'-CAAAGATGAGCAGAGAGAAATTGCT-3

siMETTL16, target sequence: 5'-GGAGCAACCTTGAATGGCTGGTATT-3

siWTAP, target sequence: 5'-GACTAGCAACCAAGGAACAAGAGAT-3

siKIAA1429, target sequence: 5'-CAAAGAAGCCTTGGGTGATTCCAAA-3

siRBM15, target sequence: 5'-GAGAGAGAAAGAGACTACCCGTTCT-3

siZC3H13, target sequence: 5'-GAAGCGCTATAGAAATGAA-3

Table S1. The clinicopathological characteristics of 183 nasopharyngeal carcinoma specimens

Parameters	Number of cases (%)
**Gender**	
Female	35 (19.1)
Male	148 (80.9)
**Age (years)**	
> 50	67 (36.6)
≤50	116 (63.4)
**WHO Pathological type**	
II	1 (0.5)
III	182 (99.5)
**T classification**	
T1-2	38 (20.8)
T3-4	145 (79.2)
**N classification**	
N0-1	87 (47.5)
N2-3	96 (52.5)
**Clinical stage**	
I-II	20 (10.9)
III-IV	163 (89.1)
**IGF2BP3 expression**	
Low	79 (43.2)
High	104 (56.8)
**6-year Tumor recurrence**	
No	155 (84.7)
Yes	28 (15.3)
**6-year Tumor metastasis**	
No	140 (76.5)
Yes	43 (23.5)
**6-year Disease progression**	
No	122 (66.7)
Yes	61 (33.3)
**6-year Vital status**	
Alive	157 (85.8)
Dead	26 (14.2)

Abbreviations: WHO, World Health Organization, II: differentiated non-keratinized carcinoma; III: undifferentiated non-keratinized carcinoma; T, tumor; N, node; IGF2BP3, insulin-like growth factor 2 mRNA-binding proteins 3

Table S2. Correlation between IGF2BP3 expression and clinicopathological characteristics of nasopharyngeal carcinoma patients

Characteristics	IGF2BP3 expression		*P* value
	Low (n=79), No. (%)	High (n=104), No. (%)	
**Gender**			
Female	17 (21.5)	18 (17.3)	0.473
Male	62 (78.5)	86 (82.7)	
**Age (years)**			
> 50	29 (36.7)	38 (36.5)	0.981
≤50	50 (63.3)	66 (63.5)	
**T stage**			
T1-2	18 (22.8)	20 (19.2)	0.557
T3-4	61 (77.2)	84 (80.8)	
**N stage**			
N0-1	47 (59.5)	40 (38.5)	0.005
N2-3	32 (40.5)	64 (61.5)	
**Clinical stage**			
I-II	13 (16.5)	7 (6.7)	0.037
III-IV	66 (83.5)	97 (93.3)	
**WHO Pathological type**			
II	1 (1.3)	0 (0.0)	0.250
III	78 (98.7)	104 (100.0)	
**Tumor recurrence**			
No	70 (88.6)	85 (81.7)	0.201
Yes	9 (11.4)	19 (18.3)	
**Tumor metastasis**			
No	70 (88.6)	70 (67.3)	0.001
Yes	9 (11.4)	34 (32.7)	
**Disease progression**			
No	65 (82.3)	57 (54.8)	<0.001
Yes	14 (17.7)	47 (45.2)	
**Vital status**			
Alive	74 (93.7)	83 (79.8)	0.008
Dead	5 (6.3)	21 (20.2)	

Abbreviations: WHO, World Health Organization, II: differentiated non-keratinized carcinoma; III: undifferentiated non-keratinized carcinoma; T, tumor; N, node; IGF2BP3, insulin-like growth factor 2 mRNA-binding proteins 3

Supplementary Figure 1

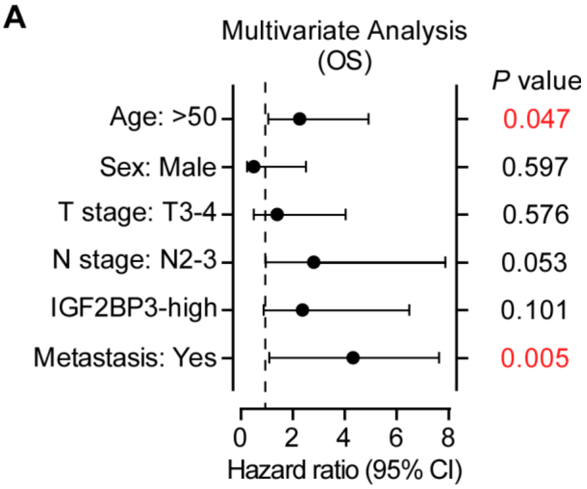

Supplementary figure 1. Multivariate Cox regression analysis of OS in the patients with NPC. (A) Multivariable Cox regression analysis to evaluate the significance of the association between high IGF2BP3 expression and OS with including metastasis as a variable.

Supplementary Figure 2

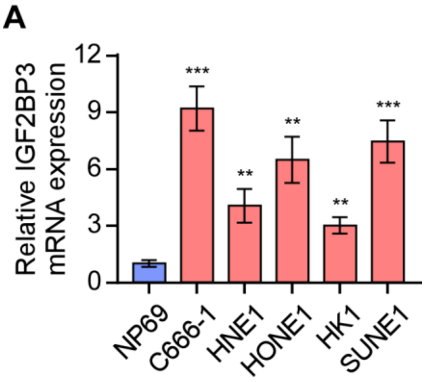

Supplementary figure 2. The mRNA expression of IGF2BP3 in NPC cell lines. (A) The mRNA expression level of IGF2BP3 in the indicated NPC cell lines. Each error bar represents the mean ± SD of three independent experiments. **, P < 0.01; ***, P < 0.001.

Supplementary Figure 3

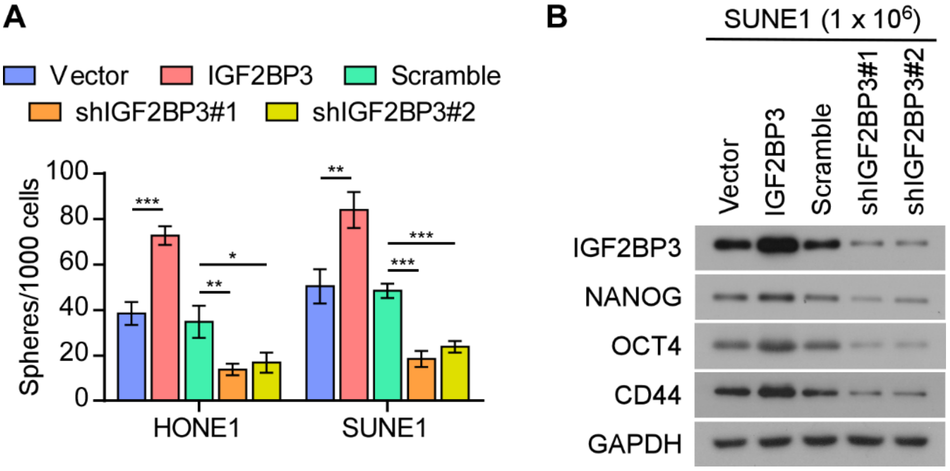

Supplementary figure 3. IGF2BP3 promotes the cancer stemness of NPC. (A) Quantification of the tumor spheres formed in the indicated HONE1 and SUNE1 cells. Each error bar represents the mean ± SD of three independent experiments. (B) Western blotting analyses of IGF2BP3 and the stemness markers (NANOG, OCT4, and CD44) in the indicated tumors. GAPDH was used as the loading control. *, $P < 0.05$; **, $P < 0.01$; ***, $P < 0.001$.

Supplementary figure 4

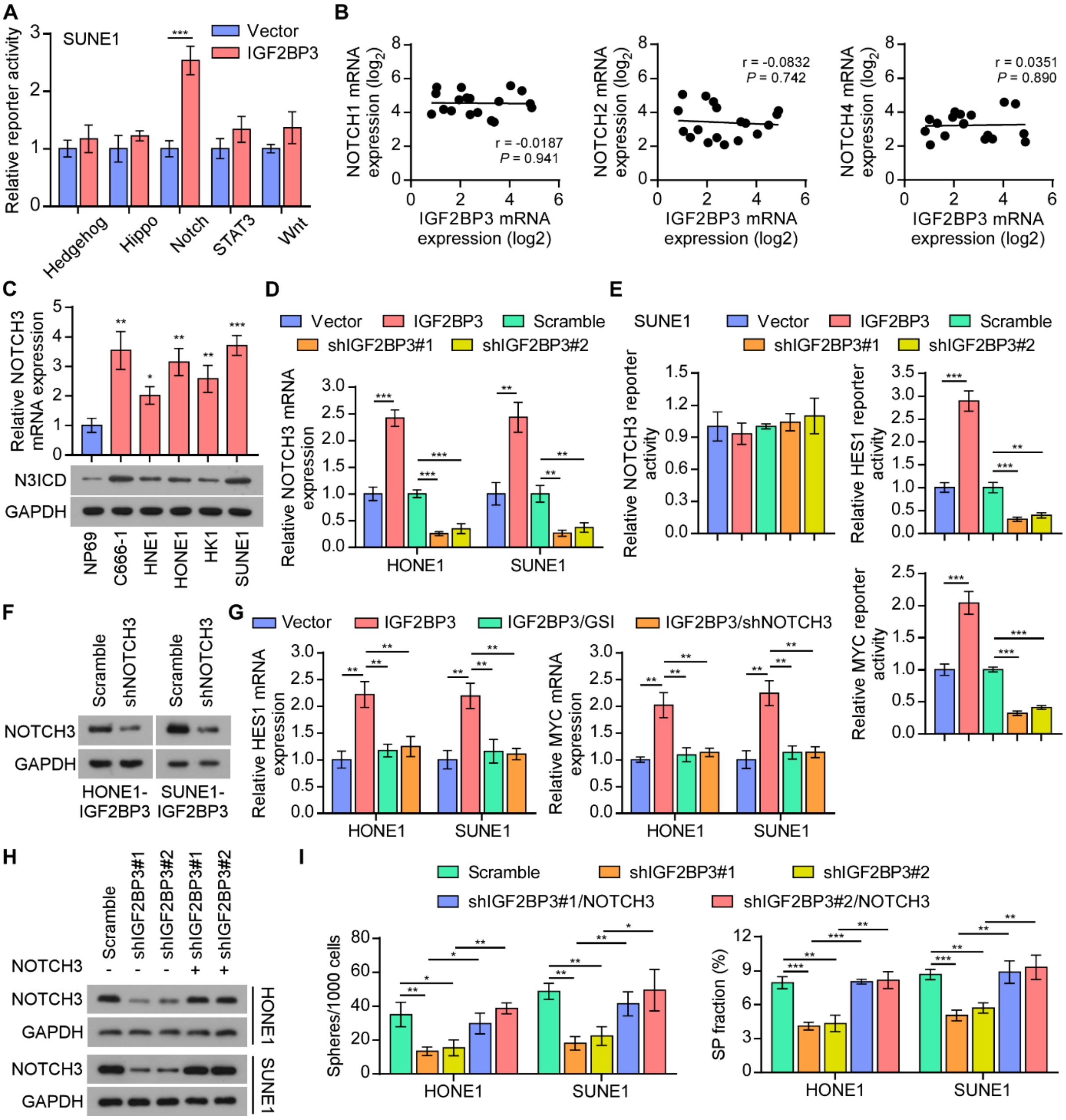

Supplementary figure 4. IGF2BP3 promoted activation of Notch3 pathway. (A) Luciferase reporter assays of Hedgehog, Hippo, Notch, STAT3, and Wnt/β-catenin reporters in SUNE1 cells. (B) Correlation between IGF2BP3 and NOTCH1, NOTCH2, NOTCH4 mRNA expression in RNA-seq data. (C) The mRNA expression level of IGF2BP3 in the indicated NPC cells. (D) qRT-PCR analysis of NOTCH3 mRNA in the indicated HONE1 and SUNE1 cells. (E) NOTCH3, HES1 and MYC luciferase reporter activity was analyzed in the indicated SUNE1 cells. (F) The protein levels of NOTCH3 in the indicated cells were detected by western blotting. GAPDH was used as the loading control. (G) qRT-PCR analysis of HES1 and MYC mRNA in indicated NPC cells. (H) The protein

levels of NOTCH3 in the indicated cells were detected by western blotting. (I) Quantification of sphere formation (left) and the side-population cells (right) among the indicated cells. Each error bar represents the mean ± SD of three independent experiments. *, $P < 0.05$; **, $P < 0.01$; ***, $P < 0.001$.

Supplementary Figure 5

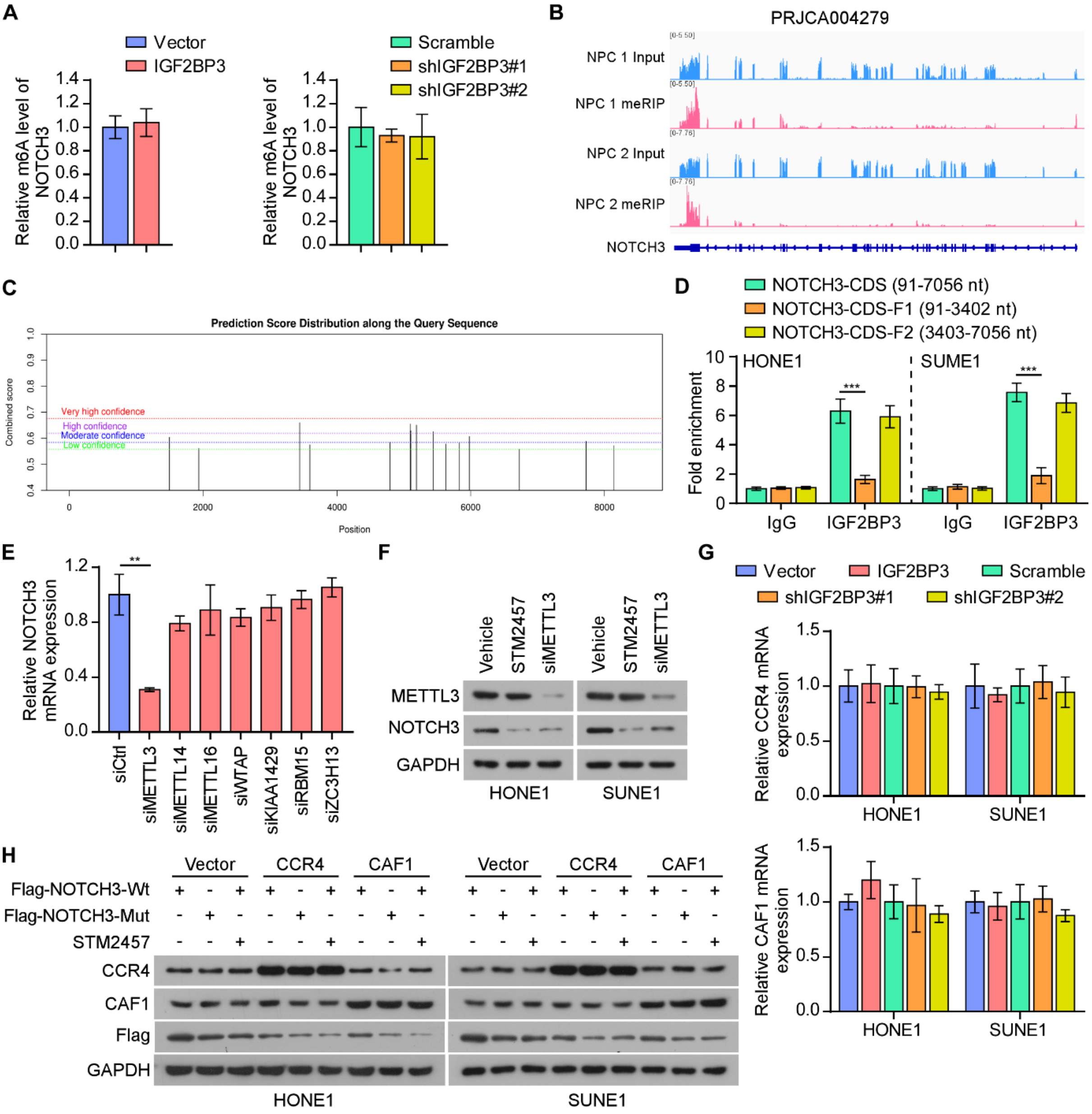

Supplementary figure 5. IGF2BP3 acts as an m6A reader. (A) Relative m6A level of NOTCH3 mRNA in indicated SUNE1 cells. (B) Analysis of m6A-modificated site on NOTCH3 mRNA in National Genomics Data Center (NGDC) datasets (PRJCA004279). (C) The prediction of m6A modification sites on the NOTCH3 transcripts RNA sequences by SRAMP. (D) RIP-qPCR assays show the enrichment of RNA fragment on IgG and IGF2BP3 in NPC cells transfected with the indicated plasmids. (E) qRT-PCR analysis of NOTCH3 mRNA in SUNE1 cells with silencing different m6A methyltransferase. (F) The protein levels of METTL3 and NOTCH3 in the indicated cells were detected by western blotting. GAPDH was used as the loading control. (G) qRT-PCR

analysis of CCR4 and CAF1 mRNA levels in indicated NPC cells. (H) The protein levels of CCR4, CAF1 and Flag in the indicated cells were detected by western blotting. GAPDH was used as the loading control. Each error bar represents the mean ± SD of three independent experiments. **, $P < 0.01$; ***, $P < 0.001$.
