# Supplementary material for: The m6A reader IGF2BP3 preserves NOTCH3 mRNA stability to sustain Notch3 signaling and promote tumor metastasis in nasopharyngeal carcinoma
Source: Oncogene. 2023 Oct 18;42(48):3564–74. doi: 10.1038/s41388-023-02865-6 (PMC10673713; doi:10.1038/s41388-023-02865-6)

Figure 1B

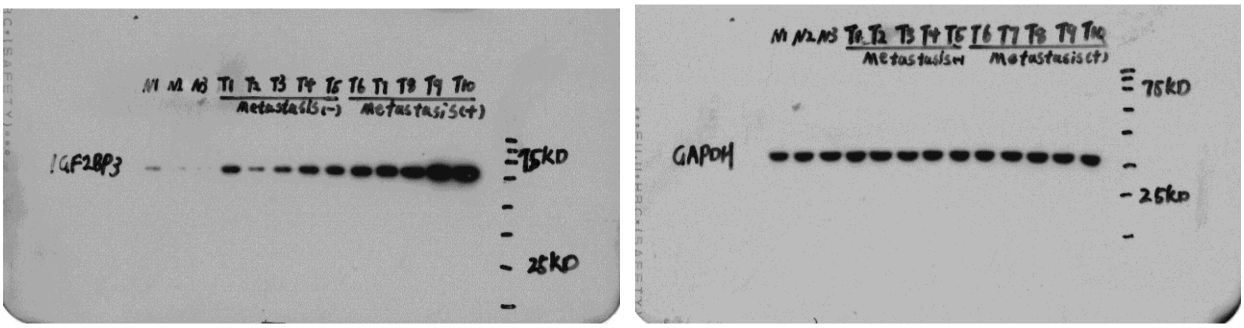

Figure 2A

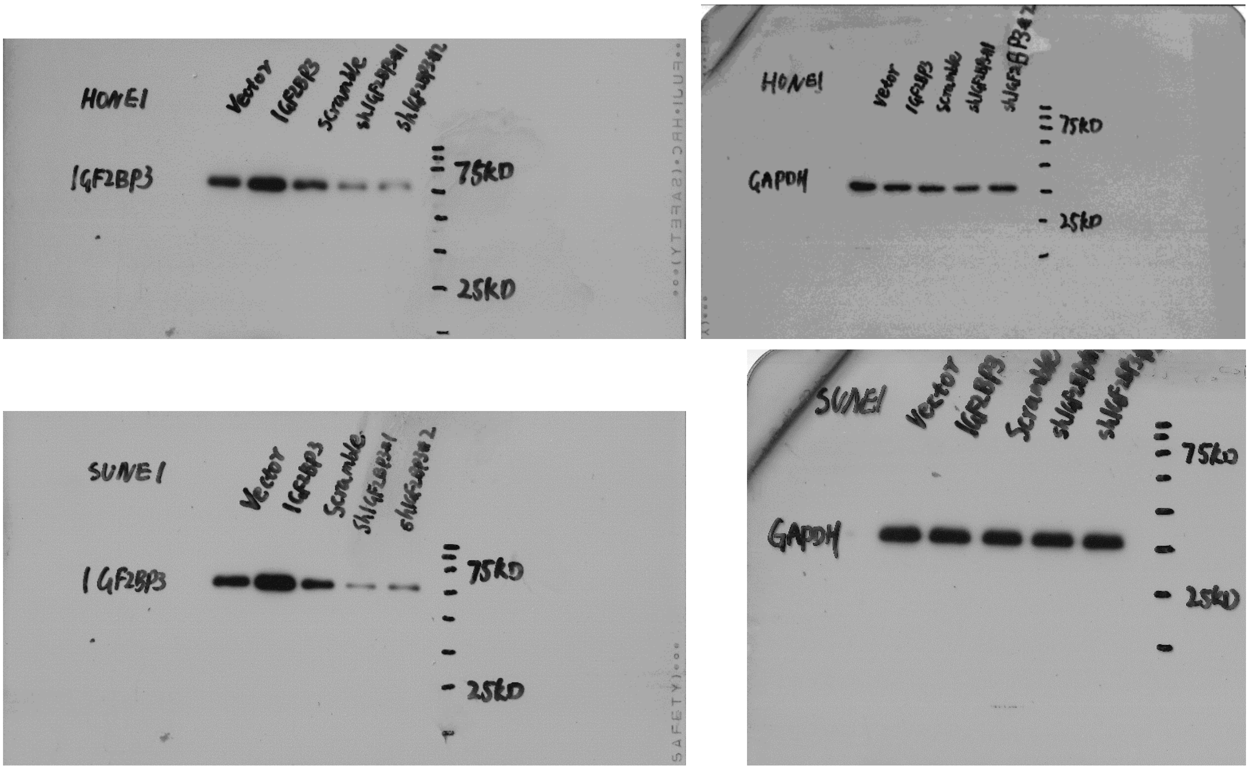

Figure 3C

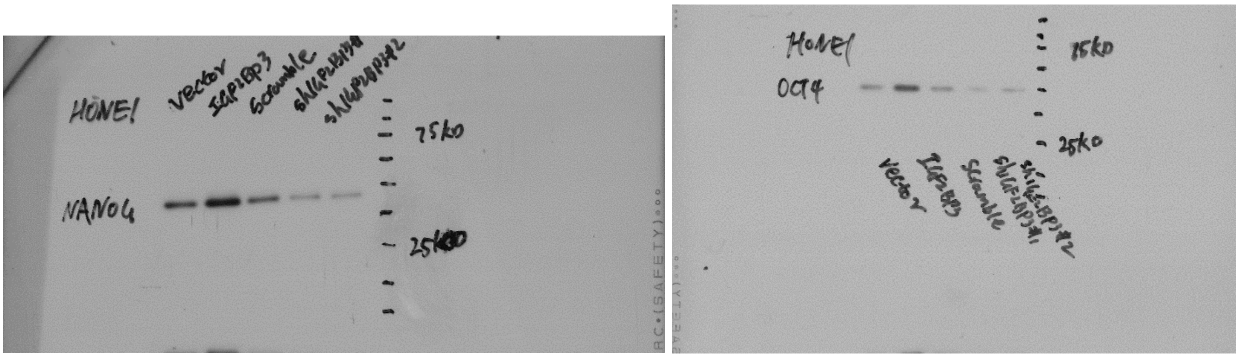

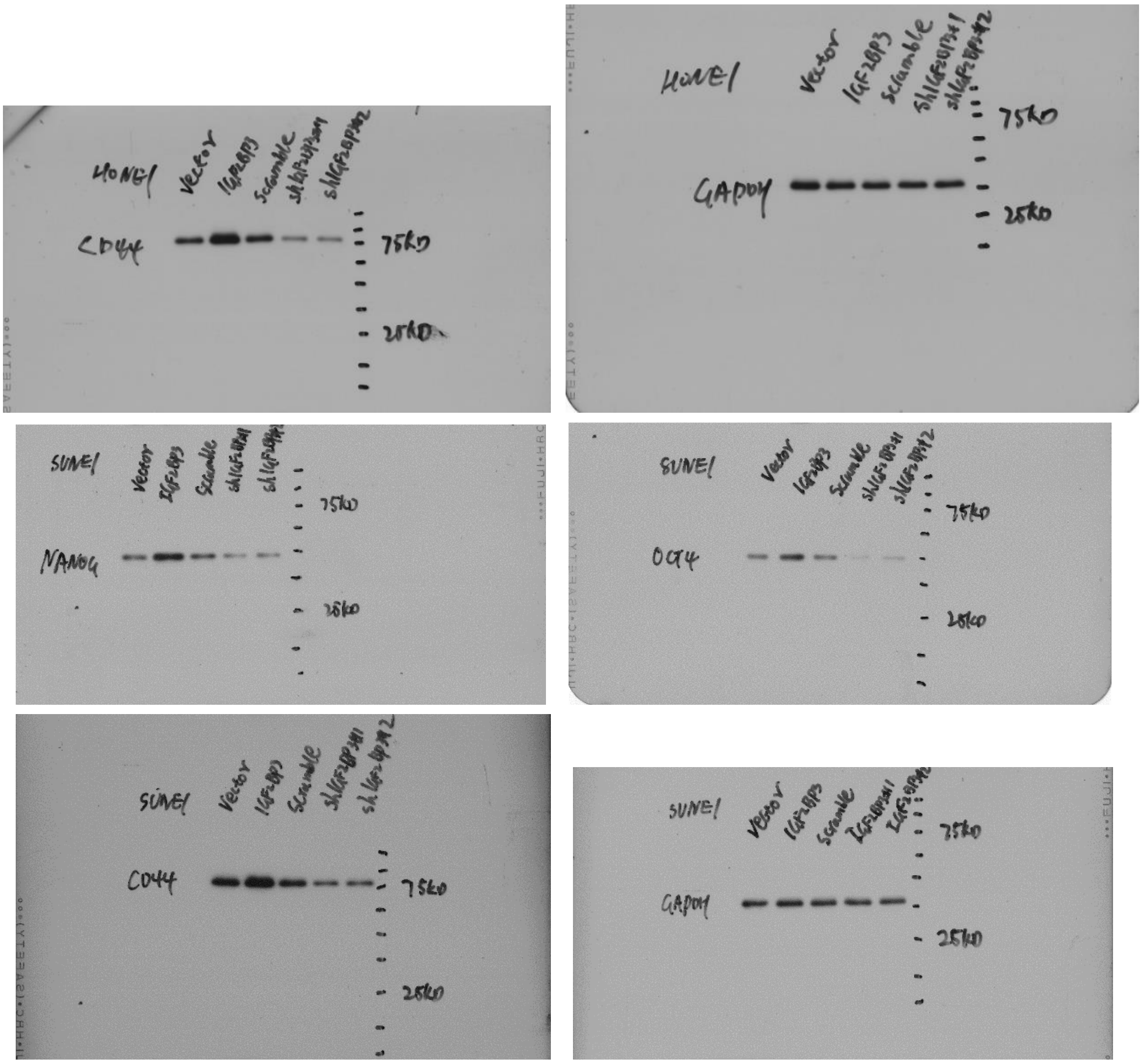
HONE1
Vector
IGF2BP3
Scramble
CD44
75kD
25kD
GAPDH
SUNE1
NANOG
OCT4

Figure 4D

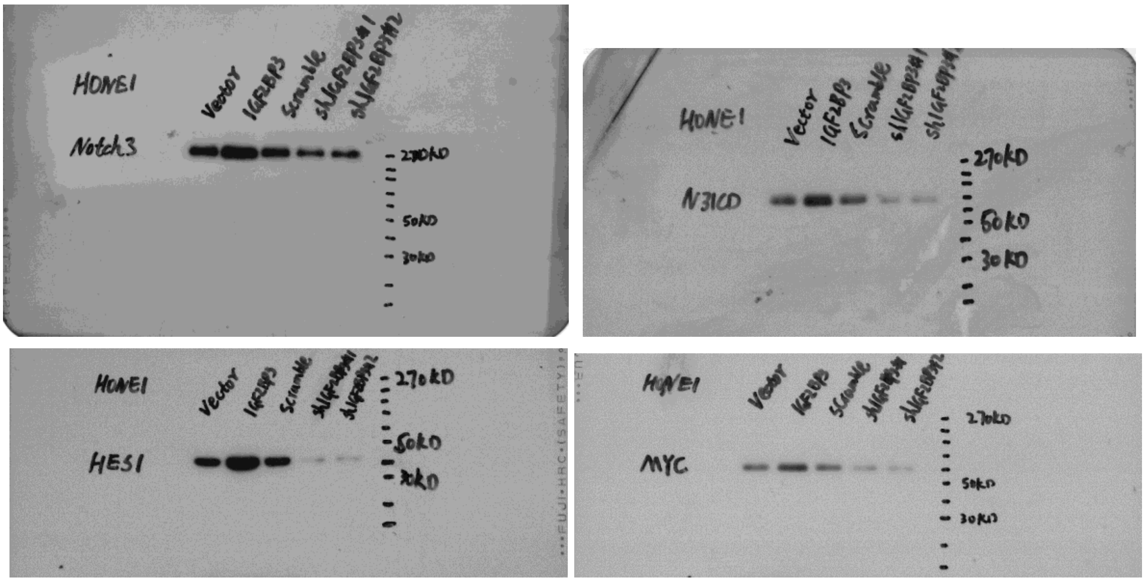
HONE1
Vector
IGF2BP3
Scramble
Notch3
N3ICD
HES1
MYC
270kD
50kD
30kD

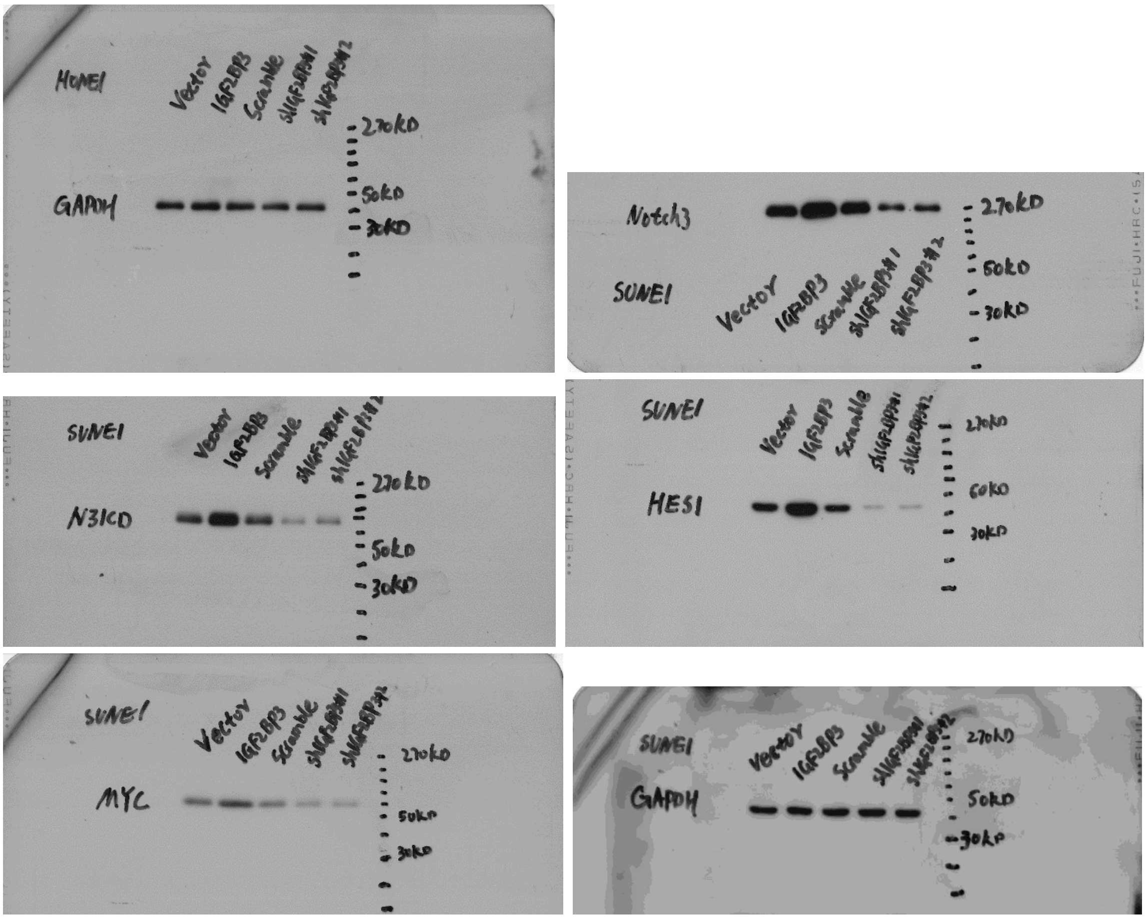
HONE1
Vector
IGF2BP3
Scramble
GAPDH
270kD
50kD
30kD
Notch3
SUNE1
N3ICD
HES1
MYC

Figure 4F

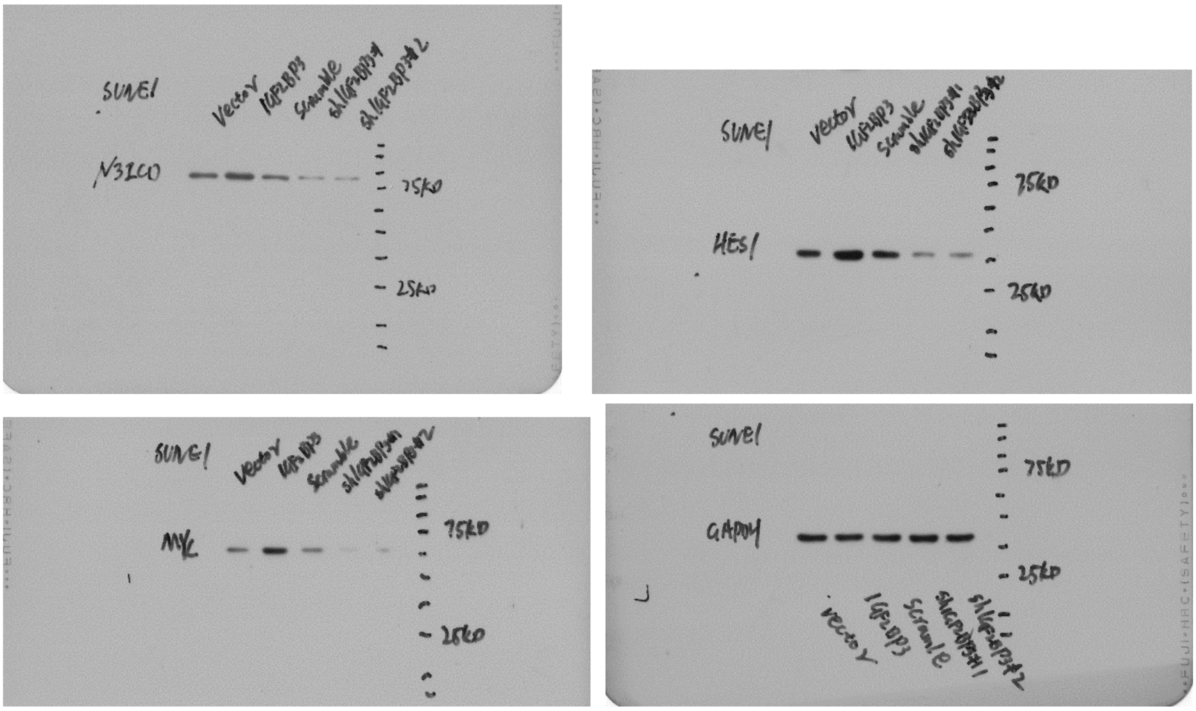
SUNE1
Vector
IGF2BP3
Scramble
N3ICD
HES1
MYC
GAPDH
75kD
25kD

Figure 6A

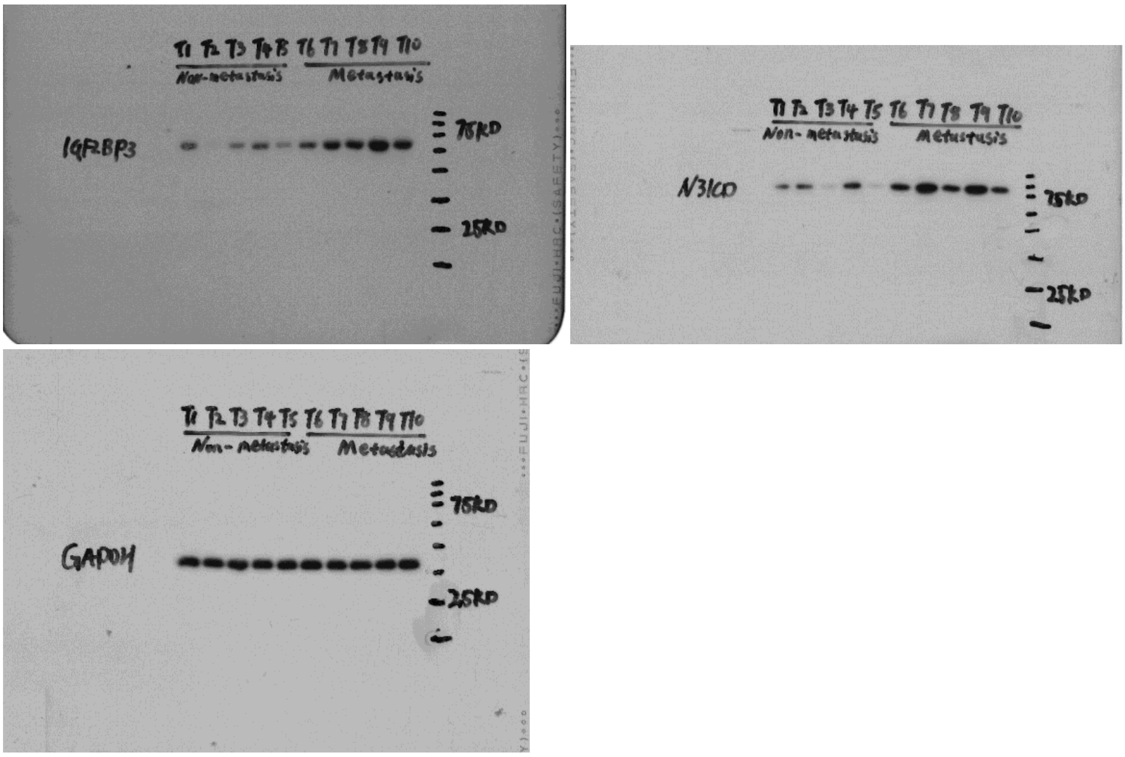

Supplementary Figure 3B

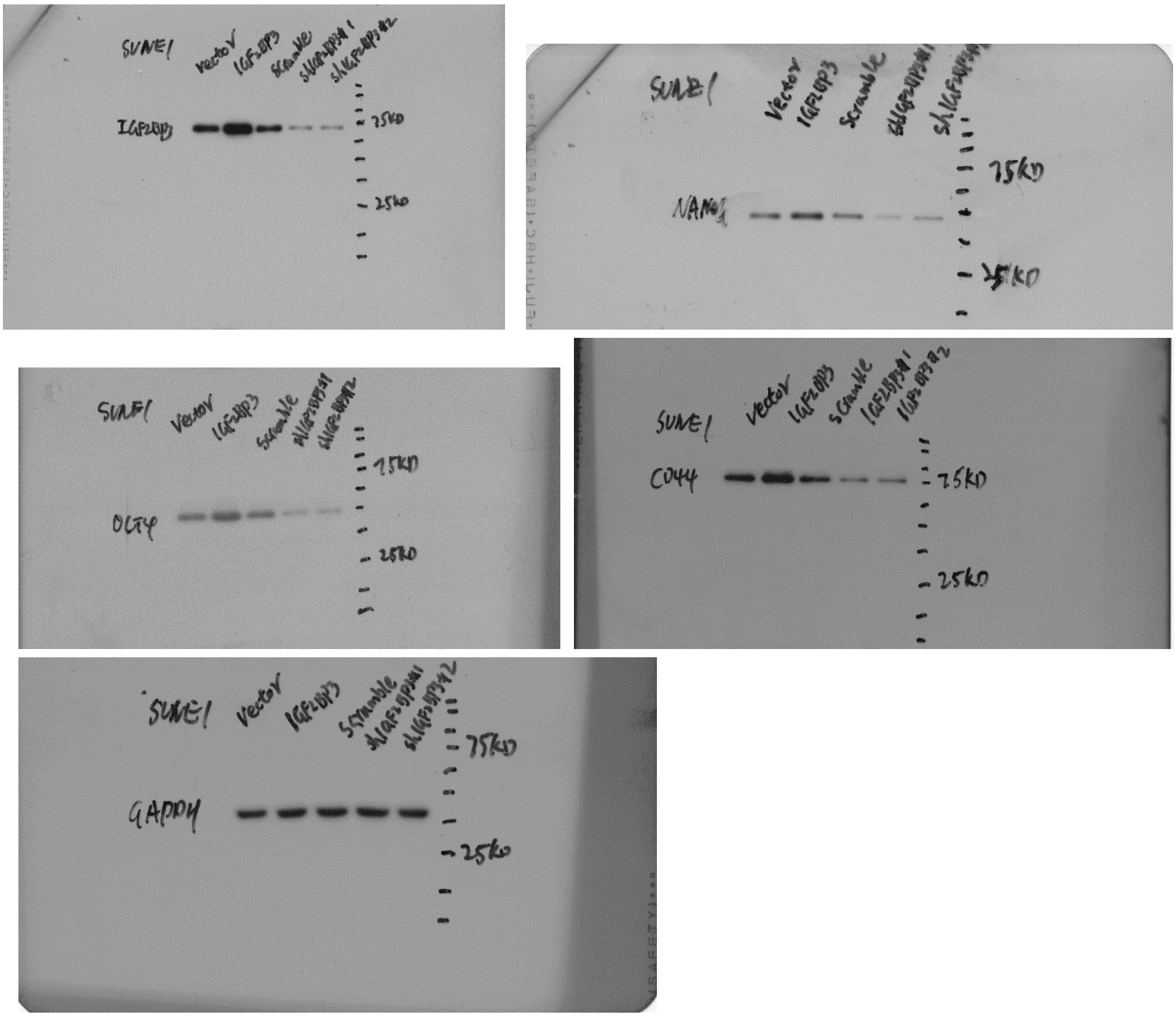

Supplementary Figure 4C

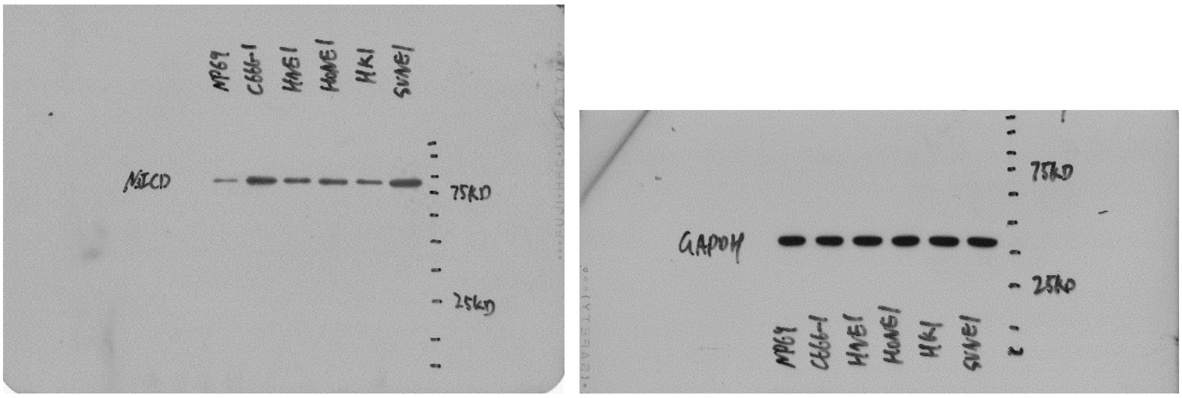

Supplementary Figure 4F

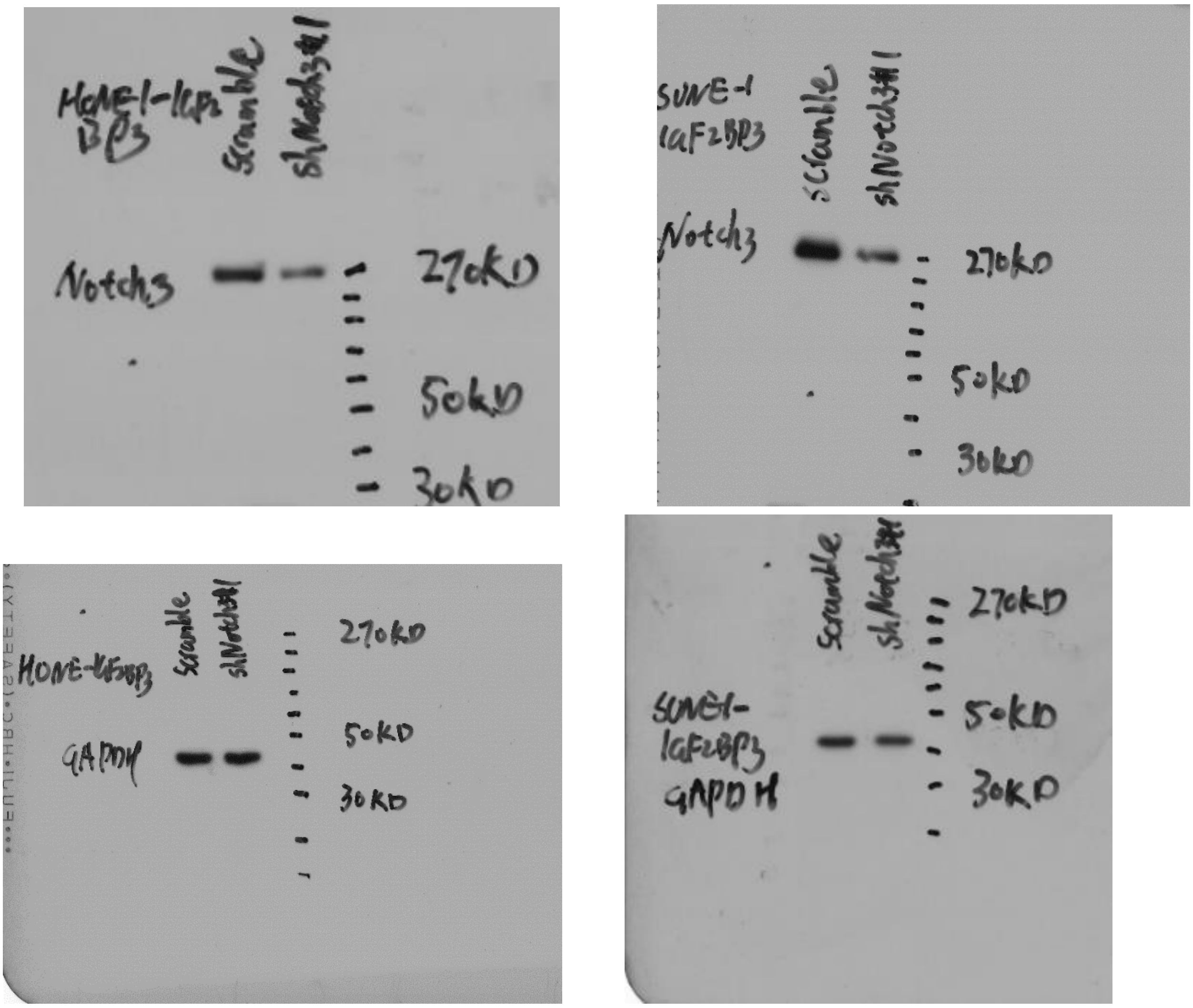

Supplementary Figure 4H

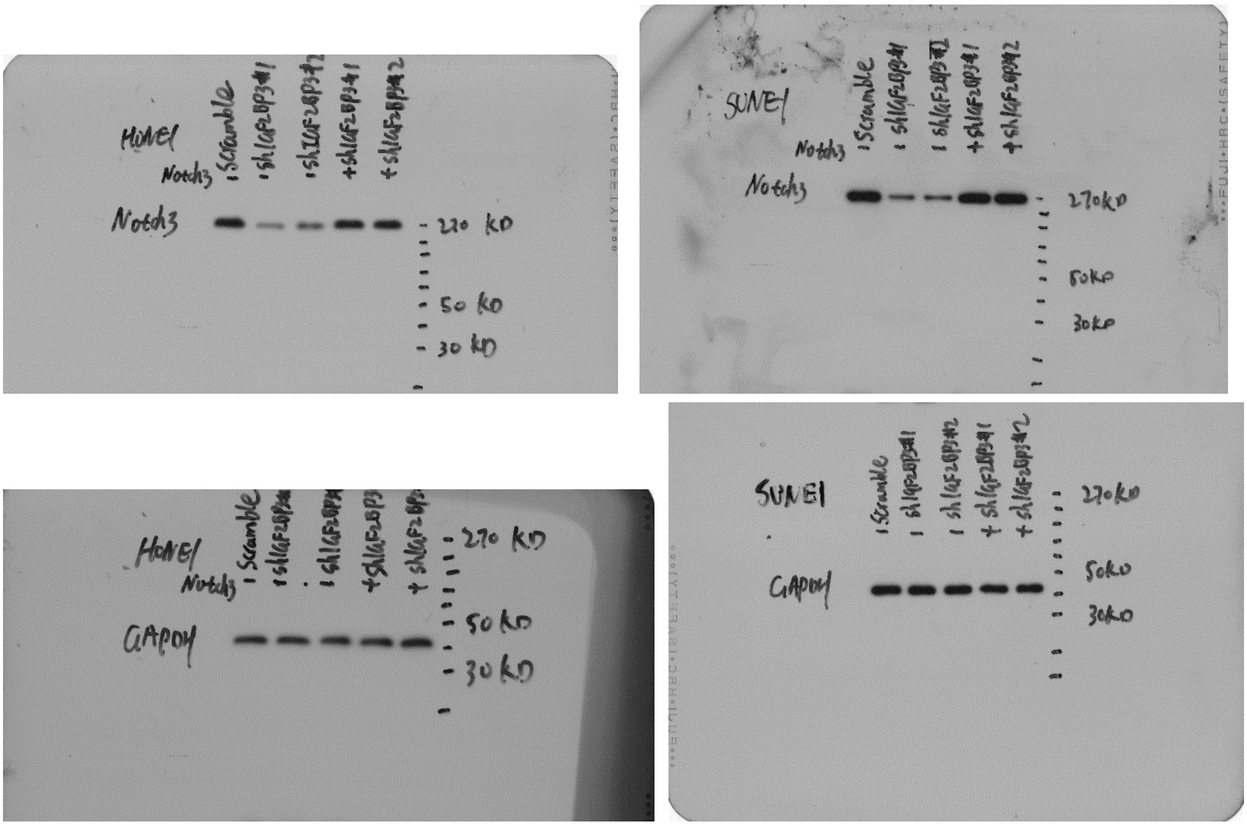

Supplementary Figure 5F

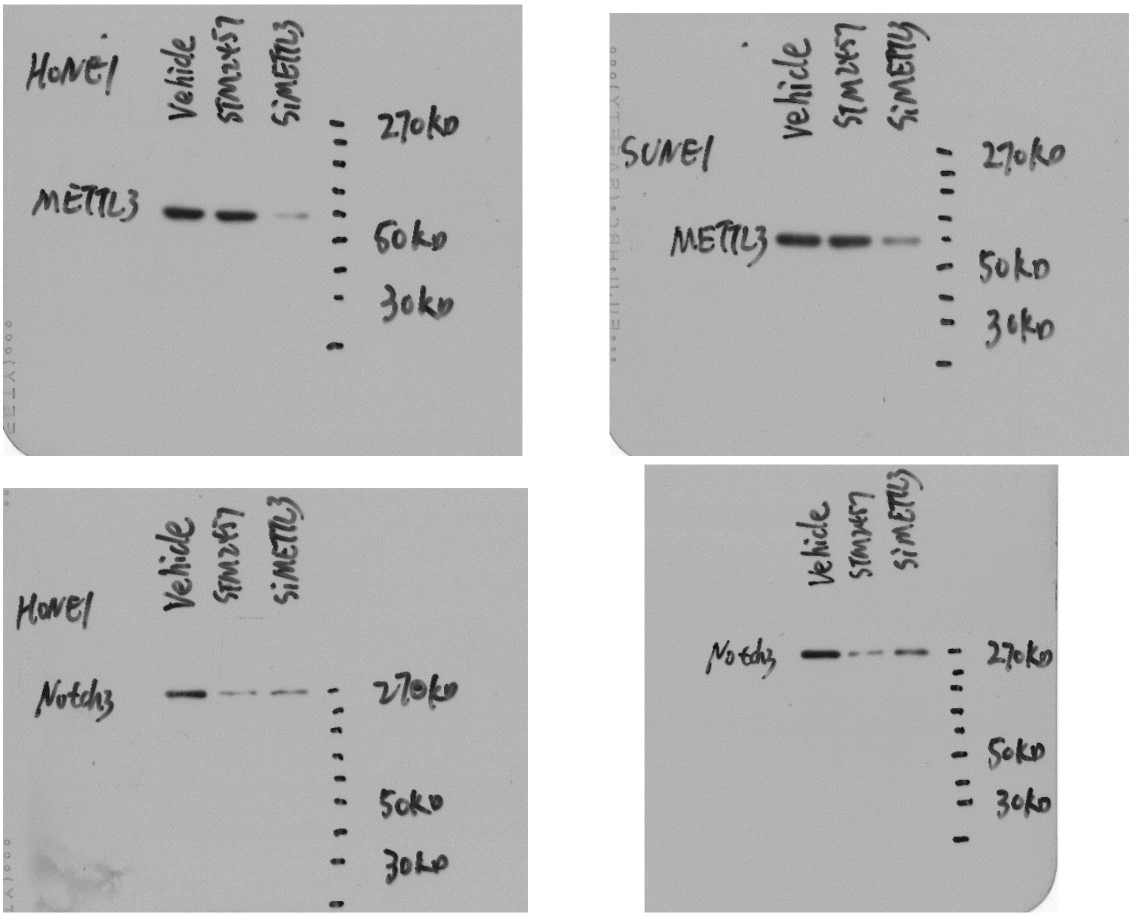

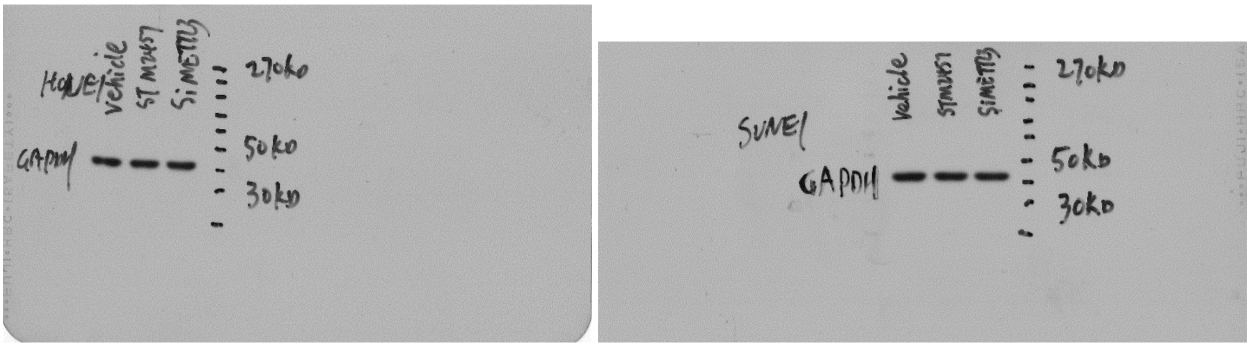

Supplementary Figure 5H

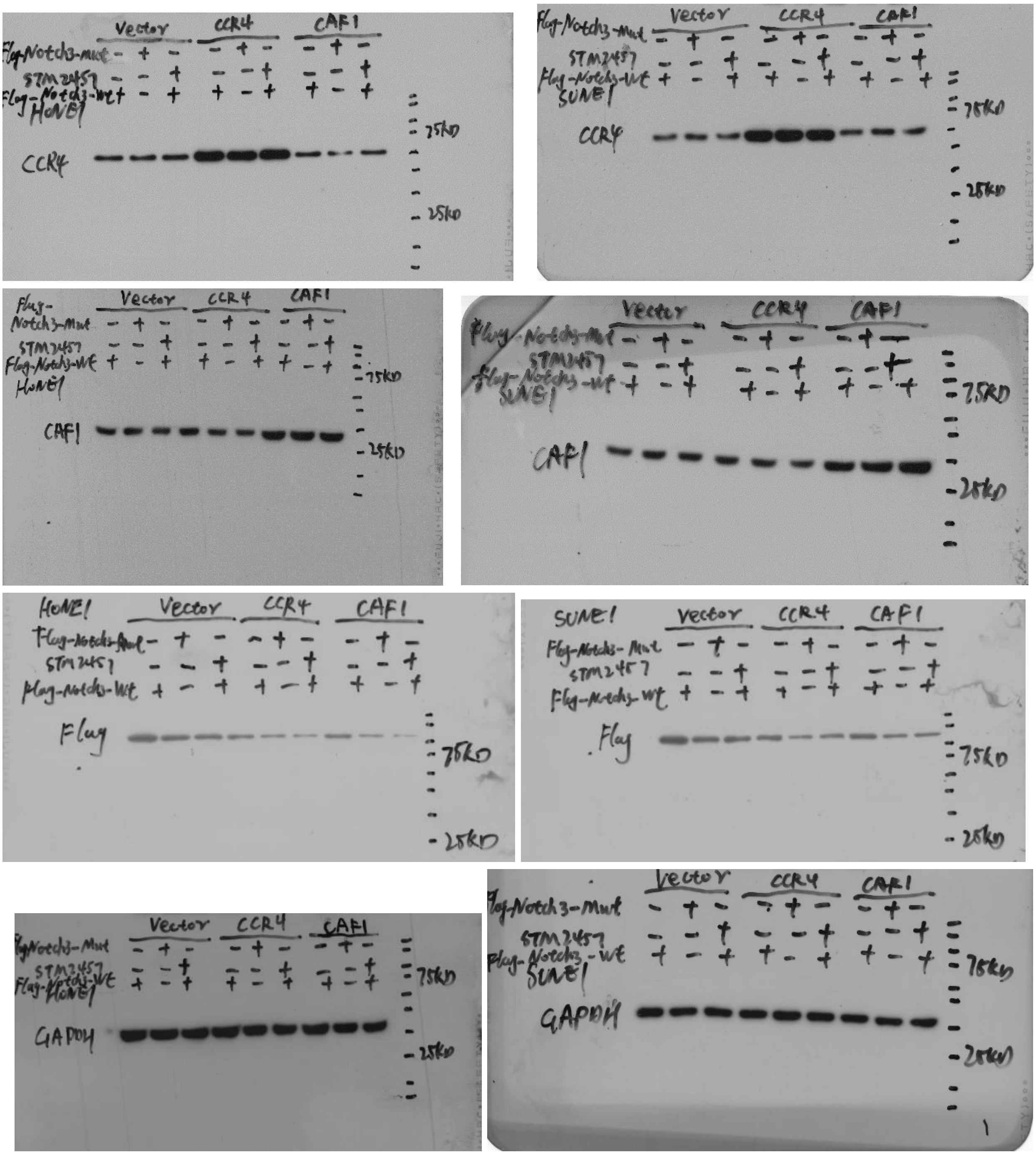

Supplement: Supplementary file 2 — Uncropped gel images [file 41388_2023_2865_MOESM2_ESM.pdf]
